# Africa’s Development Debts

**DOI:** 10.1093/jae/ejab021

**Published:** 2021-11-08

**Authors:** Benno J Ndulu, Stephen A O’Connell

**Affiliations:** 1 Mwalimu Julius Nyerere Professorial Chair on Development at the University of Dar es Salaam, Tanzania; 2 Gil and Frank Mustin Professor of Economics, Swarthmore College

**Keywords:** **JEL classification:** H63, F34, O19, H50

## Abstract

Public debt levels in sub-Saharan Africa rose sharply in the wake of the global financial crisis, and a number of countries are now classified by the World Bank and International Monetary Fund as at high risk of debt distress. By contrast with the debt crisis of the 1980s and 1990s, however, concerns were not region wide as recently as early 2020, and the policy environment for growth remains robust for the majority of countries in the region. The external environment nonetheless poses a set of region-wide risks that include the economic effects of the COVID-19 pandemic and are exacerbated by the increase in market-based debt and the retreat of the Paris Club among official creditors. Changes in perceived creditworthiness can now drive distress, and new challenges of creditor coordination will complicate the debt restructuring process. We motivate a research agenda that focuses on development assets at risk as rising debt service obligations crowd out development as well as operational and maintenance budgets. Preserving and enhancing these assets, which include advances in human capital and infrastructure and an improved investment environment, should be a central objective of domestic policy actions, preventative debt restructurings and institutional approaches to debt distress.

[I]t seems fair to call the entire Sub-Saharan region debt distressed.

Joshua Greene (1989, p. 47)

## 1. Introduction

Public debt levels in sub-Saharan Africa (SSA) rose dramatically in the wake of the global financial crisis, and by 2018 a large number of countries were classified by the World Bank and International Monetary Fund (IMF) as at high risk of debt distress. As recently as early 2020, however, debt-sustainability concerns were not region wide, in sharp contrast with the debt crisis of the 1980s and 1990s (Ncube and Brioxova, 2015; IMF, 2018a, 2019). The economic disruption from the COVID-19 pandemic now threatens to convert a severe but selective debt-management challenge into a generalised debt crisis, as countries across the region confront the exogenous fiscal shock of slower-growing revenues and elevated domestic spending requirements.

**Figure 1:**
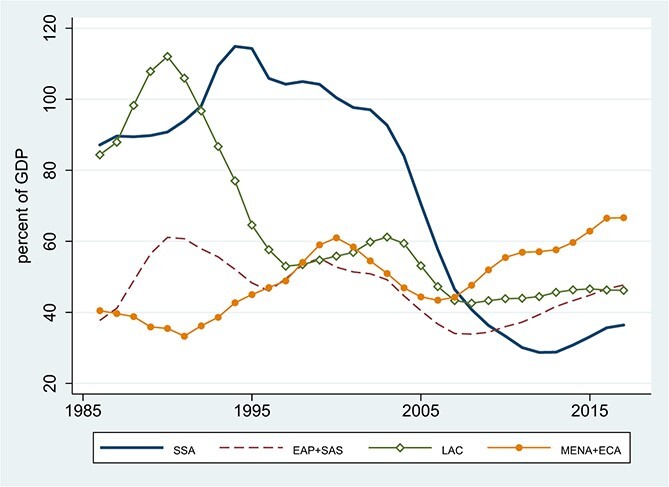
Total External Debt as a Percentage of GDP, by Region. Source: World Bank, International Debt Statistics and World Development Indicators online. Notes: The sample includes all developing countries in the indicated regions with at least 2/3 of the annual observations over the sample. To accommodate missing values, the figures shows 3-year centered moving averages of estimated time effects from panel regressions with time and country fixed effects. If all countries had the full range of observations, these time effects would just be the year-by-year cross-country averages. The regions are: SSA = Sub-Saharan Africa (44 countries, 94.9% data availability); EAP+SAS = East Asia and Pacific + South Asia (24 countries, 93.3% availability), LAC = Latin America and Caribbean (22 countries, 99.5% availability); MENA+ECA = Middle East and North Africa + Europe and Central Asia (31 countries, 87.1% availability).

**Figure 2:**
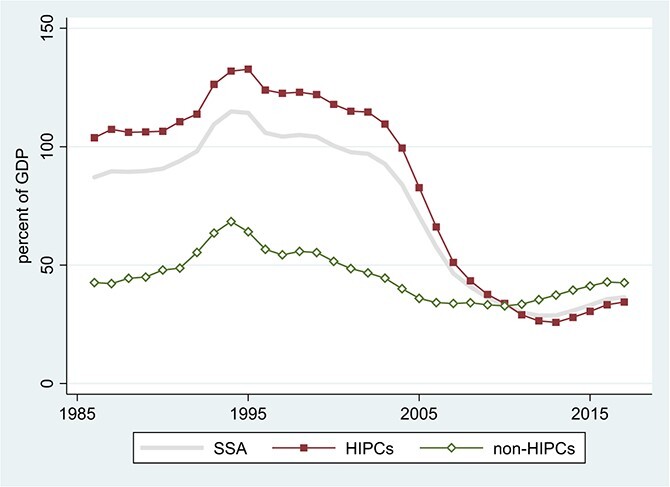
Total External Debt as Percentage of GDP in SSA. Source: World Bank, International Debt Statistics and World Development Indicators online. Notes: Three-year centered moving averages of estimated time effects in unbalanced samples; for details, see note to Figure 1. SSA = Sub-Saharan Africa (44 countries, 94.9% data availability); HIPCs = Highly Indebted Poor Countries in SSA (33 countries, 93.1% data availability); non-HIPCs = non-HIPC countries in SSA (11 countries, 100% data availability).

Figure 1 places SSA’s external debt into perspective across developing-country regions and over time. Africa is not alone in its recent surge in debt, suggesting the importance of common global drivers.^1^ Nor does the recent surge appear alarming, by comparison with the extraordinary levels of the 1980s and early 1990s. But by the late 1980s, Africa’s debts were widely viewed as unsustainable (Greene, 1989). Starting in the late 1990s, a substantial portion of their long-run decline reflects the *ex post* conversion of debts into grants, via debt-stock relief from official creditors. As shown in Figure 2, the low-income recipients of the 1996 Highly Indebted Poor Countries (HIPC) Initiative (comprising 33 of the 44 countries in SSA for which we have data) saw the largest debt reductions, enjoying successively more generous terms under the Enhanced HIPC Initiative in 1999 and the Multilateral Debt Relief Initiative (MDRI) in 2006.

The re-accumulation of debt in SSA within a decade of the HIPC/MDRI process has led some observers to the view (well in advance of the COVID-19 shock) that old-style debt problems are once again systemic in the region and that their re-emergence confirms a simple narrative about moral hazard and chronic public over-borrowing (Cropley, 2015; The Economist, 2018). Africa’s contemporary debt-management challenges differ in two important ways, however, from those of the past. First, as we will show, the re-accumulation of public debt over the course of the 2000s was preceded by deep policy reforms and accompanied by investment in human capital and public infrastructure. Africa’s debts are therefore development debts, backed by development assets. These assets include hard-fought improvements in the investment environment and are at risk if debt-servicing problems are poorly managed.

**Figure 3:**
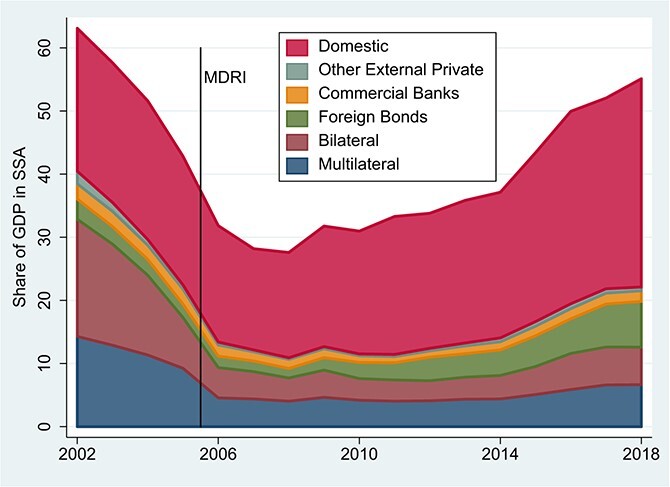
Sub-Saharan Africa: Government Debt Liabilities as a Share of GDP. Source: IMF, World Economic Outlook online, October 2019; World Bank, World Bank, International Debt Statistics online. Notes: This Figure is modeled on Figure 1.13 in IMF (2019). To construct it we added an estimate of general government domestic debt to public and publicly guaranteed (PPG) external debt. GDP in US dollars and total general government debt as a share of GDP are from IMF, World Economic Outlook. The components of PPG external debt are from World Bank, International Debt Statistics online. Domestic debt is estimated as a residual, equal to total general government debt from the IMF minus total general government external debt from the World Bank. The sample comprises the 41 countries with complete data for the period. Equatorial Guinea, South Sudan and Seychelles are excluded due to incomplete data.

Second, the profile of Africa’s creditors has changed substantially since the HIPC/MDRI period, bringing in a new set of risks associated with creditor behavior. Figure 3 shows the breakdown of public-sector debt by creditor since 2002 for the SSA region as a whole. Most striking is the large and increasing share of domestic-currency debt. Governments have relied increasingly on local-currency borrowing, predominantly in the form of bonds held by local residents. This development provides flexibility, as governments not on hard currency pegs have the option of retiring domestic debts through inflation. But for that reason, creditors typically demand a higher real return on domestic currency than on foreign-currency debt, making it an expensive option (Buffie *et al.*, 2012).

Figure 3 also reveals striking changes in the structure of Africa’s external debt. The average grant element in new external commitments remains substantial, at 23% in 2018 for all 44 borrowers with data and 27% for the 33 HIPC countries.^2^ But an increasing share of external debt is at commercial terms and held by private creditors. These flows have mainly taken the form of international debt securities. By comparison with official claims, private bond claims are shorter term, with currently obligated repayments peaking in the mid-2020s (Christensen and Schanz, 2018). These claims are also more subject to market perceptions, implying greater exposure to interest-rate volatility and rollover risk.

Among official creditors, the traditional bilateral donors (the members of the Paris Club) now hold only a small share of Africa’s external debt. These countries deliberately converted the bulk of their new flows into grants over the course of the HIPC/MDRI period, as part of an overall strategy designed to avoid the re-emergence of unsustainable debts. China, by contrast—not a member of the Paris Club—has emerged into prominence over the course of the 2000s. Chinese lending activity is non-transparent as a matter of policy and may be sharply underestimated in the global debt data reflected in Figure 3 (Horn, *et al*. 2019; Dreher *et al.*, 2017). As we will see, detailed IMF data suggest that among low-income countries in SSA, liabilities to China had by 2016 fully offset the decline in debt obligations to the traditional bilateral lenders. Chinese flows tend to be considerably less concessional on average than those of the Paris Club (Dreher *et al.*, 2017). Finally, the multilaterals retain an important role as external lenders, but not nearly as dominant a one as in the 1980s and 1990s given the entry of new lenders and the exit of their closest bilateral partners.

These developments create new debt vulnerabilities in Africa, in part by widening the gap between the shortened maturities of new debt and the long gestation periods of the economic and social infrastructure investment this debt finances. This mismatch not only creates a risk of illiquidity and default in the face of creditor inaction, but also engenders pressure for macroeconomic policy mismanagement. The proliferation of creditors, meanwhile, complicates both the credit-monitoring role of the multilaterals and the IMF’s role in coordinating creditor behavior. The annual debt-sustainability assessments that are at the core of the IMF and World Bank’s debt monitoring now affect not only the relationships between the multilaterals and African governments but also the behavior of private creditors, both foreign and domestic. The IMF, meanwhile, remains the world’s closest approximation to a debt-restructuring institution and will have to manage that role among the low-income borrowers as it has done among emerging-market borrowers. Problems of creditor coordination can delay the resolution of distress or even precipitate distress, by paralyzing the capability of creditors to provide short-term liquidity relief. These complications now include the need to replace what has arguably been a stable *modus operandi* between the Fund and its Paris Club counterparts with an effective form of coordination between China and other lenders, both official and private.

We argue in this paper that preserving and enhancing Africa’s development assets should be a focal point of debt management, both in terms of avoiding debt distress and in ensuring orderly workouts in a crisis. Throughout the paper, we identify areas where research can contribute to the successful management of Africa’s development debts. We focus particularly on low-income Africa, defined as the 39 countries with access to the most concessional resources of the World Bank and IMF.^3^ Among these countries, the official debt relief of the 2000s provides a provocative backdrop to recent developments, and the annual joint debt-sustainability exercises of the World Bank and IMF provide a rich source of detailed information. Many of our arguments apply with equal force, however, to emerging-market countries like Nigeria and South Africa.

The paper is organised as follows. In Section 2, we introduce the joint World Bank/IMF debt sustainability framework for low-income countries and interpret the evolution of debt-sustainability ratings since 2013. In Section 3, we use a simple model of over-borrowing to interpret real-time debates over multilateral debt relief and frame an overview of macroeconomic management and performance outcomes since the mid-1990s. Section 4 illustrates the use of debt-sustainability accounting to identify proximate drivers of the recent debt accumulation and draws out selected implications for debt management going forward. In Section 5, we examine the implications of a multiple-creditor world for the role of the multilaterals as grant-makers, senior lenders and risk monitors. Finally, in Section 6, we consider the state-of-the-art in debt-sustainability analysis (DSA) and introduce the concept of development assets at risk. We conclude the paper in Section 7 with a summary of the key research questions to be tackled.

## 2. Tracking debt sustainability: The LIC debt sustainability framework

We begin this section with the rudiments of DSA. We then introduce the joint IMF/World Bank debt sustainability framework for low-income countries and summarise its operation since 2005. Focusing on the deterioration of debt-sustainability ratings since 2013, we document the increasing influence of liquidity pressures in precipitating signals of distress and the heterogeneity of country experience.

Sovereign debts are sustainable if they can expect to be repaid without interruption and without requiring a restructuring of contractual terms by lenders. Interruptions of debt servicing may be driven by *insolvency*, defined as a borrower’s inability or unwillingness to meet the present value (PV) of its contractual obligations, or by *illiquidity*, defined as the borrower’s inability or unwillingness to service obligations that are coming due in the current period. These concepts are often mapped by financial analysts to the ratios of debt to gross domestic product (GDP), exports or government revenue, as indicators of insolvency risk, and the ratios of debt service to exports or government revenue, as indicators of illiquidity risk.

The 1996 HIPC Initiative operationalised these concepts by developing sustainability thresholds for debt and debt service ratios and using these to determine both the eligibility for relief and the amount of relief to be granted.^4^ With the deeper debt reduction of the MDRI in prospect, in 2005, the World Bank and IMF introduced the debt sustainability framework for low-income countries (LIC DSF), as a forward-looking framework meant to balance the need for debt sustainability with the continuing need for net resource transfers in support of the Millennium Development Goals (MDGs) (IMF and IDA, 2004). This tension—between avoiding the excessive re-accumulation of debt obligations and supporting the development ambitions of the HIPCs—places the LIC DSF squarely within the broader missions of the World Bank and IMF and is central to understanding the evolution of its design and implementation.

**Figure 4:**
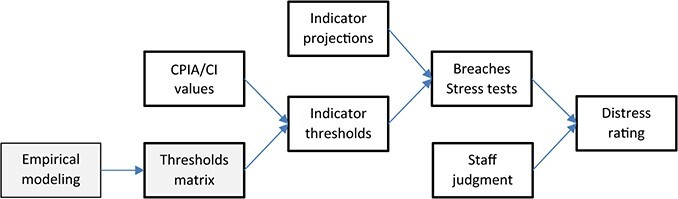
The LIC DSF (Debt Sustainability Framework). Notes: Boxes with bold outlines show the annual debt sustainability analysis (DSA) undertaken for all PRGT-eligible countries. Gray boxes show the DSF review process that occurs roughly once every 5 years. In the 2017 review, the CPIA-based assessment of debt-carrying capacity was replaced by an assessment based on a composite indicator (CI).

Indicator thresholds in the LIC DSF are derived from cross-country panel regressions that relate the indicators to past interruptions of debt service by low-income countries, with threshold levels chosen to balance the costs associated with false positives and negatives. By contrast with the HIPC thresholds, the LIC DSF thresholds are calibrated to a country’s debt-carrying capacity as measured by the quality of its economy policies and institutions.^5^ The framework is reviewed at roughly 5-year intervals, leading to intermittent changes in the thresholds and allowing for the introduction of new elements. Figure 4 shows the basic structure of the LIC DSF process.

**Figure 5:**
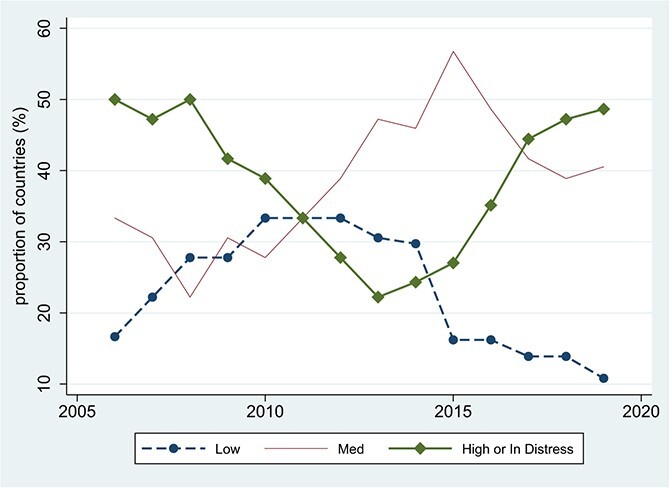
LIC DSA Risk Ratings for 37 PRGT-Eligible Countries in SSA, 2006–2019. Source: For 2006-2017, World Bank (2018); For 2018 and 2019, World Bank-IMF ``Lists of LIC DSAs for PRGT-Eligible Countries'' dated August 1, 2018, May 21, 2019, and April 30, 2020, and IMF (2020), p. 14.

Since 2006, a DSA within the LIC DSF framework has been applied on nearly an annual basis to all low-income countries eligible for the IMF’s concessional resources. Figure 5 shows that the proportion of low-income African countries assessed to be at high risk or already in distress fell from 50% in 2006 to just above 20% in 2013, and then reversed course and rose monotonically back to nearly 50% in 2019. As background to interpreting these developments, we briefly review some of the underlying debt-sustainability algebra.

At the core of any debt sustainability assessment is a stock-flow identity that relates changes in a country’s public and publicly guaranteed (PPG) debt to the country’s projected capacity to meet debt-servicing obligations through new financing or adjustments to its primary fiscal deficit. To illustrate, suppose that a government borrows externally in dollars and issues no domestic liabilities. Define ${D}_t$ as the end-of-period external debt stock measured in dollars, ${e}_t$ as the end-of-period exchange rate in local currency units per dollar, ${P}_t$ as the GDP deflator and ${Y}_t$ as real GDP. Debt accumulation then has to satisfy the government’s period-by-period budget constraint

(1) \begin{equation*} {E}_t\left({D}_t-{D}_{t-1}\right)={PDEF}_t+{E}_t{i}_t{D}_{t-1}+{V}_t, \end{equation*}

where ${PDEF}_t$ is the primary fiscal deficit net of grants, ${i}_t$ is the average nominal interest rate on external debt, ${E}_t$ is the period-average exchange rate and ${V}_t$ is an amalgam of residual influences on the debt stock, including valuation effects and debt forgiveness.

High levels of the ratio of external public debt to GDP (${d}_t\equiv{e}_t{D}_t/{P}_t{Y}_t$) are correlated with the emergence of debt-servicing difficulties in cross-country data and are widely used to signal solvency pressures. Equation (1) allows us to solve for the dynamics of the debt to GDP ratio. Ignoring the valuation term in (1)^6^, ${d}_t$ evolves according to

(2) \begin{equation*} {d}_t-{d}_{t-1}=\left[\frac{\left(1+{i}_t\right)\left(1+{\hat{e}}_t\right)}{\left(1+{\pi}_t\right)\left(1+{g}_t\right)}-1\right]{d}_{t-1}+\frac{1+{\hat{e}}_t}{1+{\hat{E}}_t}\cdot \left(\frac{PDEF_t}{P_t{Y}_t}\right), \end{equation*}

where ${\hat{e}}_t$ and ${\hat{E}}_t$ are the rates of end-of-period and period-average exchange-rate depreciation, ${\pi}_t$ is the inflation rate of the GDP deflator, ${g}_t$ is the growth rate of real GDP and $PDEF/ PY$ is the ratio of the primary fiscal deficit to GDP.

In equation (1), ${D}_t$ refers to the book value of debt. If the debt were fully non-concessional, this would be the discounted value of the associated repayment obligations, calculated at the risk-adjusted interest rate at which the borrower was able to access private markets. To incorporate the concessionality of LIC debts, the LIC DSF tracks the present values (PVs) of debt obligations rather than their book values. A version of equation (2) can of course be applied to the present value of debt; the concessionality structure of new debt commitments would then have an immediate impact on the debt ratio, as it does in the LIC DSF. In an equation like (2), non-concessional inflows would enter unchanged, adding dollar for dollar to the debt stock, while concessional inflows would combine a smaller increment to the present value of debt with a grant element that reduces the appropriate concept of the primary deficit net of grants.^7^

The ratios of debt service to exports and government revenue play an important role in the LIC DSF, by signaling sustainability risks associated with periods of illiquidity. For a given average interest rate and maturity of debt obligations, these indicators behave like their debt-stock counterparts (e.g., the ratio of the present value of external debt to exports), reflecting both the size and the concessionality of the underlying obligations. But unlike the debt ratios, the debt-service ratios are highly sensitive to the maturity structure of a country’s obligations and the irregular timing of its overall repayment schedule.

The term in square brackets in (2) is approximately ${r}_t-{g}_t,$ where ${r}_t\equiv{i}_t+{\hat{e}}_t-{\pi}_t$ is the country’s real interest rate (in this example, on foreign borrowing). When the interest rate exceeds the growth rate, this dynamic equation is unstable. For given values of the parameters including the fiscal stance as summarised by $PDEF/ PY,$ a debt stock that is rising continues to rise, and by a larger amount each year. Unless the relationship between the real interest rate and the real growth rate reverses—and especially if the approach of debt distress raises borrowing costs—a primary surplus is required to prevent explosive debt dynamics.

Buiter (1985) and others use (2) to explore paths of the primary balance and debt under alternative assumptions about the country’s capacity for fiscal adjustment. When the real interest rate exceeds the real growth rate, sociopolitical constraints that limit the pace or magnitude of fiscal adjustment can generate a dramatic form of unsustainability in which the debt path is predicted to explode despite ongoing fiscal adjustment efforts. The Buiter approach is incorporated into a sophisticated general equilibrium model of public infrastructure investment by Buffie *et al.* (2012), an important paper to which we will return in a later section.

Table 1 shows the evolution of debt and debt-service thresholds in the LIC DSF system. Developments in the table reflect adjustments to the framework resulting from the 5-year reviews. The 2009 reforms left the 2005 thresholds in place but introduced an adjustment for countries with large inward remittances, to reflect their greater debt-carrying capacity. Threshold values for debt service to revenue were then reduced considerably in the 2012 LIC DSF reforms (implemented in 2013) and further tightened when the thresholds for debt service to exports and total debt to GDP were reduced in the 2017 reforms (implemented in 2018). The 2017 LIC DSF reforms also replaced the CPIA-based risk filter with a composite indicator that combines the CPIA with a macroeconomic assessment that gives weight to real GDP growth, remittances, international reserves and global growth. Taken together, the reforms implemented in 2013 and 2018 have increased the sensitivity of the framework to liquidity-based pressures.

**Table 1:** Selected Indicator Thresholds in the LIC DSF

CPIA- or CI-based capacity	PV of external PPG debt/GDP			Debt service/exports			Debt service/revenue			PV of total PPG debt/GDP		
	2005	2013	2018	2005	2013	2018	2005	2013	2018	2005	2013	2018
Weak	30	30	30	15	15	10	25	18	14	—	38	35
Medium	40	40	40	20	20	15	30	20	18	—	56	55
Strong	50	50	55	25	25	21	35	22	23	—	74	70

*Source:* IMF (2012, 2013, 2017).

*Note:* Years indicate when the thresholds were first implemented. Thresholds for the external debt-to-exports and debt-to-revenue ratios are omitted from the table. To place these thresholds into context, the 1996 HIPC Initiative targeted a debt service to export ratio of 20–25 and a debt-to-exports ratio of 200–250. The 1999 Enhanced HIPC Initiative reduced these to 15–20 and 150. In 2005, the LIC DSF thresholds for the ratio of the PV of PPG external debt to exports were 100, 150 and 200.

The 2013 and 2018 thresholds were implemented as a result of LIC DSF reviews in 2012 and 2017. The PV of total PPG debt indicator became mandatory for the first time following the 2012 review. Following the 2009 review (starting in 2010), the framework included remittances in the denominators of the external debt to GDP, external debt to exports and external debt service to exports ratios and assigned remittance-adjusted thresholds (not shown). In 2013, the remittance-adjusted thresholds were set at 10%, 20% and 20%, respectively, below the unadjusted thresholds that appear in the table above.

Until 2018, the debt-carrying capacity assessment (along the rows) was based on a 3-year backward moving average of the overall CPIA score (which ranges from 1 to 6). Values of 3.25 or below, between 3.25 and 3.75 or 3.75 or above corresponded to weak, medium and strong. This classification was replaced in July 2018 by a composite indicator (CI) that includes the CPIA and four macroeconomic variables, with weak, medium and strong mapping to the lower, medium two and upper quartiles of the historical distribution of the CI.

For countries not already ‘In distress’ (experiencing a serious interruption in debt repayments), the LIC DSF ratings in Figure 5 are derived by using equations like (2) to project the debt and debt-service indicators for given fiscal policy settings and alternative assumptions about shocks. Projected indicator values are compared with the pre-set thresholds in Table 1.^8^ Indicator breaches that occur in the baseline or stress-test scenarios constitute the mechanical signals in the framework and map the country to provisional low-, medium- or high-risk status. Staff judgment then plays a role in determining the final classification, which can differ from the classification based on the mechanical signals alone.^9^

The deteriorating risk assessments in Figure 5 therefore have a few proximate contributors. Rising debt and debt-service ratios point to the drivers identified in equations like (2): adverse developments in the relationship between the real interest rate and the constant-local-currency growth rates of GDP (and/or of exports and fiscal revenues), and large values of the primary deficit relative to GDP. For given values of the indicators, assessments can also deteriorate due to slippage in the perceived policy environment, changes in the LIC DSF thresholds and staff judgment.

**Figure 6:**
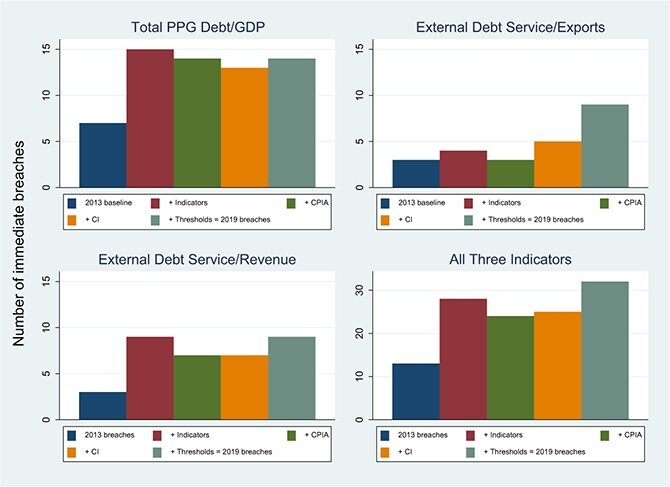
Simulation of Immediate LIC DSA Breaches, 2013 and 2019. Source: Indicator values and the thresholds appropriate to the country's debt-carrying capacity are from the most recent available IMF report for each country that includes a LIC DSA for the periods ending December 31, 2013 and December 31, 2019. Notes: The Figure includes the 35 PRGT-eligible countries in SSA with DSAs available for both years. Bars 2-5 show the simulated impact on total immediate breaches of [bar 2] changes between 2013 and 2019 in the indicators alone; [bar 3] changes in the indicators and the CPIA; [bar 4] changes in the indicators and the CPIA, and also the 2018 switch from the CPIA to the CI; and finally [bar 4] all changes including the 2017 change in indicator thresholds.

In Figure 6 below we explore the relative contributions of indicator deterioration, reduced carrying capacity and modifications in the threshold matrix, in accounting for the deterioration in risk ratings in SSA between 2013 and 2019. Drawing on the debt-sustainability analyses published in IMF country documents, we focus on what we call *immediate breaches*, defined as breaches that are present in the initial projection year of the relevant DSA (in most cases, 2013 or 2019). The first and last bars in each panel of Figure 6 show the number of immediate breaches in 2013 and 2019. All three indicators show a sharp increase in breaches, consistent with deterioration in overall risk ratings between the 2 years. The two debt service indicators display the sharpest relative deterioration, implying that liquidity pressures played an increasing role in signaling debt distress over this period.

The intermediate bars unpack these developments into the effect of deterioration in the indicators, deterioration in assessed debt-carrying capacities and changes in the LIC DSF thresholds. Deterioration in the indicators (the difference between the first and second bars) explains the entire increase in the debt to GDP and debt service to revenue signals. The indicator values play almost no role, by contrast, in accounting for the end-to-end increase in breaches from the debt service to export ratio. This suggests that reduced growth in the nominal dollar values of GDP and government revenues played a more important role in explaining the deterioration of overall risk ratings over this period than did deterioration in the repayment terms on new loans, despite the overall shift towards non-concessional forms of borrowing shown earlier in Figure 3.

The third and fourth bars in Figure 6 show the impact of changes in the debt-carrying capacity assessment between the 2 years. We unpack this effect into reassessments that would have occurred due solely to changes in the CPIA, and reassessments associated with the shift in 2018 from a CPIA-based assessment to a CI-based assessment. Neither of these developments had much impact on the total number of immediate breaches or the relative importance of the three indicators. These results are unsurprising; the African LICs have remained clustered within the ‘Weak’ CPIA category (overall CPIA $\le$3.25) and the shift from CPIA to CI was almost certainly implemented with a view to avoiding immediate reclassifications of debt-carrying capacity.

Finally, the difference between the fourth and fifth bars shows the impact on breaches of changes in the threshold matrix that were implemented in 2018. For the total debt to GDP and debt service to revenue indicators, this impact was small. The debt service to export indicator is an exception: the thresholds for this indicator were substantially tightened as a result of the 2017 LIC DSF review (Table 1), and this tightening accounts for most of the increase in debt service to export breaches between 2013 and 2019. A similar impact may well have been created by the tightening of debt service to revenue thresholds following the 2012 LIC DSF review, but these thresholds were implemented in 2013, so uncovering their impact would require an earlier baseline year.

## 3. Déjà vu? Distress, relief and new borrowing, 1980–2019

Table 2 shows the full set of country transitions in the LIC DSF rating system between 2013 and 2019, with deteriorations appearing in the upper right triangle of cells. The latter group is large and heterogeneous, consisting of primary-commodity exporters and diversified exporters, fragile and non-fragile countries and frontier markets along with countries less integrated into global financial markets. The breadth of this development, barely two decades from the Jubilee 2000 Campaign and the launch of the HIPC Initiative in 1996, has created a strong sense of déjà vu among some observers.

**Table 2:** LIC DSA Risk-of-Debt-Distress Transitions in SSA, 2013–2019

Transitions from row to column		2019			
		Low	Moderate	High	In distress
2013	Low	Senegal^dm^, Tanzania^dm^, Uganda^dm^	Benin^dp^, **Kenya**^dm^, Liberia^df^, Madagascar^df^	Cameroon^df^, Ethiopia^dp^, Zambia^cm^	Congo, Rep.^cf^
	Moderate	Rwanda^dp^	Burkina Faso^cp^, Cote d’Ivoire^dfm^, Guinea^cf^, Guinea-Bissau^cf^, **Lesotho**^dp^, Malawi^cf^, Mali^cf^, Niger^cp^, Togo^df^	**Cabo Verde** ^d^, CAR^cf^, Ghana^dm^, Mauritania^cp^, Sierra Leone^cf^	The Gambia^df^, Mozambique^cm^, South Sudan^cf*^
	High		Comoros^df^, Congo Dem. Rep.^cf^	Burundi^cf^, Chad^cf^	Sao Tome and Principe^df^
	In distress				Eritrea^cf*^, Sudan^cf^, Zimbabwe^cf^

*Source:* For 2013, IMF (2018a), Table 3 and Annex Table 1 and IMF country reports; for 2019, World Bank (2018), Table 4; IMF, List of LIC DSAs for PRGT-Eligible Countries, May 21, 2019; and IMF (2020) ‘Evolution of Debt Vulnerabilities in Lower Income Economies’, Table 1.

*Note:* Non-HIPC countries are shown in bold type. South Sudan’s first year of LIC DSA classification was 2014. Eritrea’s most recent LIC DSA was 2009. The superscripts classify countries as either commodity exporters (c) or diversified exporters (d) and as either frontier markets (m), developing markets (p) or fragile states (f). Cote d’Ivoire is both a frontier market and a fragile state. The classification comes from IMF (2018), Annex Table 1, and omits Cabo Verde, which is PRGT-eligible but not categorised as a low-income country by the IMF. Using the IMF’s criteria, Cabo Verde is a diversified exporter and is not a fragile state; its classification as a developing or frontier market is unclear and we omit it.

We will argue in this section that the differences between Africa’s recent debt accumulation and its pre-HIPC accumulation are more important than the similarities. To frame this debate we briefly describe the HIPC and MDRI initiatives and then examine an argument about over-borrowing that was well known to the architects of the HIPC Initiative. Easterly (1999, 2002) argued that unless debt cancellation was accompanied by changes in the policy environment—an outcome he saw little evidence to support in previous instances of debt relief in SSA—debt problems would re-emerge and lenders would end up facing a new round of distress.

### 3.1 HIPC and MDRI

By the mid-1980s the level and ongoing dynamics of debt in SSA made it clear that despite the intermittent debt relief efforts of the bilateral creditors, most countries were either failing to service their debts or at very high risk of distress (Krumm, 1985; Greene, 1989; Humphreys and Underwood, 1989). The multilateral creditors had been prevented by their charters from joining earlier debt relief efforts, and therefore held an increasing share of the growing debt. The 1996 HIPC Initiative recognised the need to cancel multilateral debts outright, with parallel reductions provided by the members of the Paris Club of bilateral creditors.

The target of the HIPC Initiative was to reduce debt ratios to sustainable levels, with the sustainability targets made more generous in the Enhanced HIPC Initiative of 1999. Introduced in 2005, the MDRI made much deeper debt stock reduction available, starting in January 2006, to countries that had qualified for debt-stock relief under the HIPC Initiative. The objective of the MDRI was to create space for increases in fiscal spending related to the Millennium Development Goals (Cassimon *et al.*, 2015).

**Figure 7:**
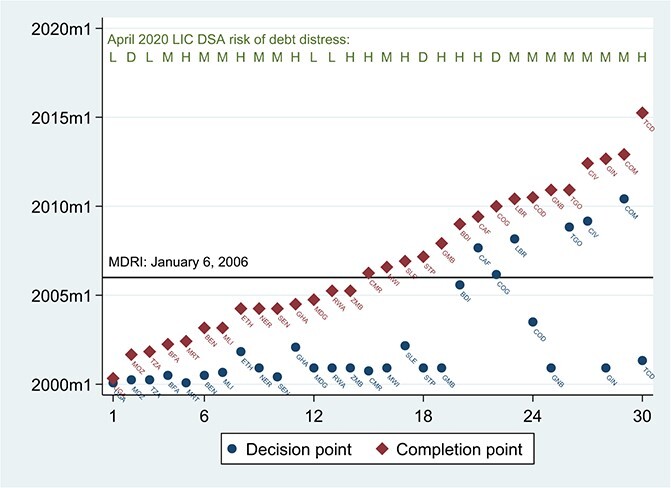
HIPC Decision and Completion Points, SSA. Source: Decision and Completion points are from World Bank and IMF (2018). Eritrea, Somalia and Sudan had not reached the Decision Point as of June 2020. The debt distress ratings are from the LIC DSAs as reported by the World Bank and IMF for April 30, 2020. The first round of MDRI debt relief was delivered on January 6, 2006, to the 19 HIPCs that had reached the Completion Point.

HIPC debt relief was conditional. To reach the ‘Decision Point’ and qualify for immediate debt service relief, countries had to have an IMF program in good standing. To reach the ‘Completion Point’ and receive irrevocable debt stock relief, countries had to develop and implement a national poverty-reduction strategy and satisfy other triggers (Cassimon *et al.*, 2015). Figure 7 shows the timing of HIPC Decision and Completion points for the 33 HIPC countries in SSA. All 14 countries in SSA that had reached the Completion point by the end of 2005 received their MDRI relief in January of 2006. Starting in 2005, all HIPC countries were subject to annual debt-sustainability reviews under the LIC DSF.

### 3.2 Over-borrowing and futile debt relief

Equation (2) provides a basis for skepticism regarding the impact of debt relief. In the presence of a primary deficit (net of grants), cutting ${d}_t$ in half will place the debt ratio on a permanently lower path, other things equal. But if $r>g,$ that path will be rising. Unless other parameters in the equation change, debt will be re-accumulated and the country will eventually need a new round of debt relief.

**Figure 8:**
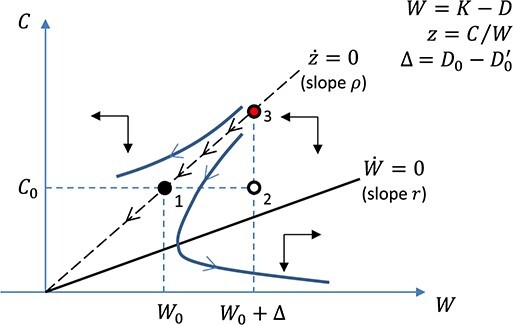
‘Futile’ Debt Stock Relief in Easterly (1999). Note: The country is at point 1 when it receives debt cancellation that reduces the present value of its debt stock from $D_0$ to $D_0^{\prime}=D_0-\Delta$. National wealth jumps to $W_0+\Delta$, and consumption jumps to point 3.

Easterly (1999) formalised this observation by arguing that debt relief would be futile if the discount rate applied to intertemporal decisions by African policy elites was far above not only the cost of borrowing but also the return to domestic investment. Easterly’s analysis appears in Figure 8, where the aggregate production function takes the simple form $Y= AK$ for a broad concept of $K$ that includes human capital, public infrastructure and private physical capital, and where Easterly eliminates a potentially crucial role of borrowing in the development process by assuming that the real return to capital is equal to the real interest rate on external debt $(A=r)$. National net worth in this simple framework is given by $W=K-D.$ Real GDP is $AK= rK,$ and real gross national product (netting out interest payments to foreign lenders) is $rW=r(K-D)$. The country chooses its consumption and investment pattern in order to maximise an intertemporal welfare function that is defined over national consumption, discounting future utility at the rate $\rho >r.$

The dynamic equation for consumption in this model is the familiar Euler equation

(3) \begin{equation*} \frac{{\dot{C}}_t}{C_t}=\sigma \left({r}_t-\rho \right), \end{equation*}

where $\dot{C}=d{C}_t/ dt$ and $\sigma >0$ is the intertemporal elasticity of substitution. With $\rho >r,$ the country’s impatience to consume drives it to deplete its net worth over time, leading to continuous declines in consumption and net worth. The path of external debt in this analysis is determined exogenously by what official lenders are willing to provide. To fix ideas, we assume that the present value of accumulated debt obligations is equal to ${D}_0$ and has been fixed for some time. The country then arrives at point 1 from a point like 3, with the capital stock and GDP falling and the debt to GDP ratio continuously rising. Regardless of the particular history of borrowing—including its concessionality^10^—the country has become highly indebted.

Debt-stock relief, in this context, provides an instantaneous jump in national net worth by an amount equal to the present value of the cancelled obligations ($\Delta$ in Figure 4), leaving the economy momentarily at point 2. But there is no change in the incentive to invest, and so the relief is fully dissipated in a consumption boom. Consumption jumps immediately to point 3, where consumption and net worth resume their decline from a higher level, reproducing the initial situation in due time. Debt limits, moreover, have no effect in this context, because if the providers of debt relief were to prevent future borrowing in order to avoid the pressure to cancel new debts, the country would simply run down its assets.

Easterly (1999, 2002) provided empirical evidence that the debt relief provided by bilateral creditors to the HIPC countries in the two decades preceding the HIPC Initiative had failed to improve the environment for investment and growth. The HIPC Initiative, he concluded, was unlikely to do better. This was in part a rejection of the debt overhang literature, which had argued that the emergence of external arrears created a Marshallian inefficiency by converting unpaid debts into a form of equity. The lingering claims of unpaid creditors, by this argument, exerted the equivalent of a tax on investment and policy effort, because lenders could stake a claim to any growth in available resources.

But Easterly’s over-borrowing argument was at bottom a characterisation of the political economy of policy in SSA. In this respect his conclusions were a product of the time: his data sample ran from 1980 to 1997, a period of market-contrary policy regimes in SSA. As we emphasise below, however, the 1990s were the cusp of what would later prove to be a decisive improvement in the policy environment across the region (Ndulu *et al.*, 2007, 2008; O’Connell and Dolan, 2012). The Easterly over-borrowing story has traction in some individual cases, but it is inconsistent with the broad evolution of policy and economic performance over the HIPC/MDRI period.

Easterly’s argument was one of two debt sustainability concerns that motivated the architects of official debt relief for low-income countries and the LIC DSF regime. The second was a concern that debt relief would stimulate irresponsible new lending, either by the multilaterals themselves or by third-party lenders. With respect to multilateral and Paris Club lending, the LIC DSF was one of a number of institutional safeguards designed to maintain a high degree of *ex ante* concessionality in official resource flows, by shifting from loans to grants and ensuring a high grant element in new loans. In the most extreme of these accommodations, the (IDA allocation) formula used to determine the concessionality of World Bank resources was made fully contingent on a country’s LIC DSF ratings, with deterioration signaling a shift from loans to grants. Third-party lenders, in turn, were virtually absent in the early years of HIPC, so conventional concerns about the externalities to non-coordinated lending or the indirect reliance of third-party lenders on future official bailouts were not prominent in the design of HIPC. But as MDRI approached, these concerns were addressed through the non-concessional borrowing policies of the World Bank and IMF, both of which were tied directly to the LIC DSF ratings. The tension between these two objectives—maintaining net flows from official donors and preventing over-lending by third parties—was then navigated over time against the background of the Millennium Development Goals, the global financial crisis and the emergence of China and international bond markets as important new sources of finance.

### 3.3 Debt relief in retrospect

We examine the debt relief period through three lenses in this section, looking at broad macroeconomic developments, recent empirical papers on the impacts of HIPC and MDRI and case-study evidence on the diversity of experiences across the region.

**Figure 9:**
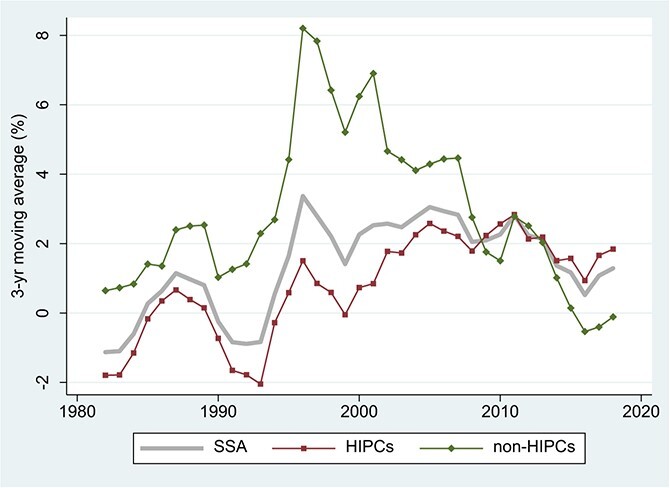
SSA: Growth in Real GDP Per Capita, 1982–2019. Source: IMF, World Economic Outlook October 2019. The sample period is 1980 to 2019. Notes: Three-year centered moving averages of estimated time effects in unbalanced samples; for details, see note to Figure 1. SSA = Sub-Saharan Africa (43 countries, 96.9% data availability); HIPCs = Highly Indebted Poor Countries in SSA (31 countries, 97.4% data availability); non-HIPCs = non-HIPC countries in SSA (12 countries, 95.7% data availability).

Easterly’s argument was that debt relief would be wasted if the policy environment for growth did not change and that there was little basis to expect it to change. What is clear in retrospect is that the policy environment for growth in SSA improved immensely both before and during the HIPC/MDRI period. Ndulu *et al.* (2007, 2008) document widespread improvements in economic policy dating from the mid-1990s and argue that these reforms created an environment more benign to investment and productivity growth than during the previous two decades. Figure 9 shows growth outcomes in SSA starting in the early 1980s. Among the HIPCs, a 3-year moving average of growth rates begins to rise in the mid-1990s and then remains above its earlier average in *every year* of the two decades that followed the announcement of the HIPC Initiative in 1996. The World Banks’s CPIA index registers a parallel improvement over these decades: for the 33 HIPCs in SSA, it rises from an average of 2.77 in 1986–95 to 3.08 in 1996–2005 and 3.21 in 2006–2015.^11^

With the benefit of hindsight, Easterly (2019) acknowledges the durability of reforms over the course of the debt relief period and their association with favorable growth outcomes. Table 3, taken from Easterly (2019), documents that the share of countries in SSA with extreme policy weaknesses was much lower in the post-HIPC period than earlier and that these reforms were strongly associated with faster growth. In a regression-based decomposition, the elimination of extreme policies (proxied by high values for the black market premium, inflation and real exchange-rate overvaluation and by much-lower-than-predicted ratios of trade to GDP) accounts for nearly 3/4 of the observed increase in per-capita real GDP growth between 1980–98 and 1999–2015. These correlations do not imply a causal path from policy to growth, but it is clear that the HIPC/MDRI period coincided with major improvements in both policy and growth.

**Table 3:** How Much Does Reform Explain Recovery From the ‘Lost Decades’ in Africa?

	Africa 1980–1998	Africa 1999–2015	Actual change in growth (%)	Predicted change in growth (%)
Per capita growth rate (%)	0.1	1.8	1.76	1.27
Frequency of policy outcomes (%):				
Black market premium >40%	27.5	3.7		0.22
…between 20% and 40%	10.8	0.7		0.04
Inflation rate >40%	14.6	3.0		0.31
…between 20% and 40%	15.3	4.9		0.13
Real interest rate <−20%	9.2	2.2		0.05
…between −20 to −5	20.1	8.9		−0.02
Overvaluation >100%	15.0	4.1		0.17
…between 50% and 100%	20.2	10.1		0.03
Residual trade share >40%	19.0	8.1		0.25
…between 30% and 40%	15.0	6.5		0.10

*Source:* Easterly (2019), Table 5a.

*Note:* The table shows the frequency of bad policy outcomes in Africa in 1980–98 and 1999–2015 and the associated changes in predicted growth, based on the coefficients from a panel regression that controls for all policies and includes country and year effects.

**Figure 10:**
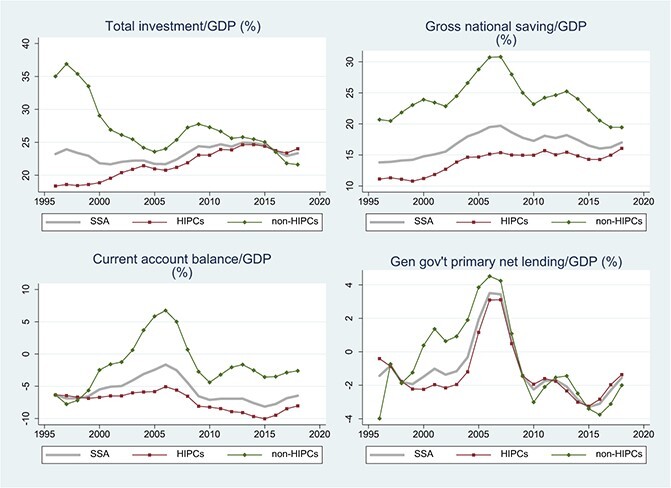
SSA: Macroeconomic Developments, 1996–2017. Source: IMF, World Economic Outlook October 2019. The sample period is 1995 to 2019. Notes: Three-year centered moving averages of estimated time effects in unbalanced samples; for details, see note to Figure 1. SSA = Sub-Saharan Africa (For investment, saving, CA balance, and Primary net lending, the sample comprises 42, 43, 45 and 45 countries, with 99.4%, 99.5%, 99.6% and 92.5% data availability); HIPCs = Highly Indebted Poor Countries in SSA (30, 31, 32, and 32 countries; 99.2%, 99.4%, 99.4% and 92.8% data availability); non-HIPCs = non-HIPC countries in SSA (12, 12, 13 and 13 countries; 100%, 100%, 100% and 92% data availability).

Figure 10 tracks broad macroeconomic aggregates since 1996 and reveals a pattern consistent with a permanent improvement in the growth environment. The HIPC group displays a steady and protracted investment boom starting around 2000, initially financed more than point-for-point by higher gross national saving and therefore accompanied by an improvement in the current account. The primary fiscal balance is steady and then sharply improving among the HIPCs in the early 2000s, moving very strongly into surplus until 2007 before reversing during the global financial crisis and its aftermath. Starting around 2005, national saving stabilises as investment continues to rise. The most recent decade is characterised by a rising current account deficit as the counterpart of increased reliance on external finance.

While the global evidence on debt-overhang effects and the impacts of debt-stock relief on the borrower country continues to be mixed^12^, recent empirical evidence on the impacts of HIPC and MDRI is largely favorable on our reading. Djimeu (2018) applies a differences-in-differences approach to investment and growth within Africa (HIPC countries versus non-HIPC countries) and finds large and statistically significant contributions of the HIPC Initiative to public investment and growth.^13^Cassimon *et al.* (2015) focus on the reductions in debt service implied by HIPC and MDRI and find that both efforts led to enhanced revenue collection, an impact at odds with the Easterly (1999, 2002) analysis. On the spending side, Cassimon *et al.* (2015) find that the HIPC Initiative raised public investment while the MDRI led to an increase in current primary (non-interest) public spending.

The impact of conditionality on these developments is of intense interest, given the continuing close relationship between the LICs and the multilateral institutions. Cassimon *et al.* (2015) argue that the post-MDRI increase in current spending suggests a loss of fiscal discipline and a return of Easterly-style dynamics due to the removal of the Completion-Point conditions for receiving MDRI relief. While this view may have traction in selected cases, the situation is complex. The stated purpose of the MDRI was to free up spending for the MDGs. This prominently included current spending devoted to education, health and the maintenance of public infrastructure, all of which have a substantial investment component and acquired a strong countercyclical motivation following the onset of the global financial crisis. The composition of spending responses is crucial in determining their alignment with a simple over-borrowing story and is an important topic for further research.

**Figure 11:**
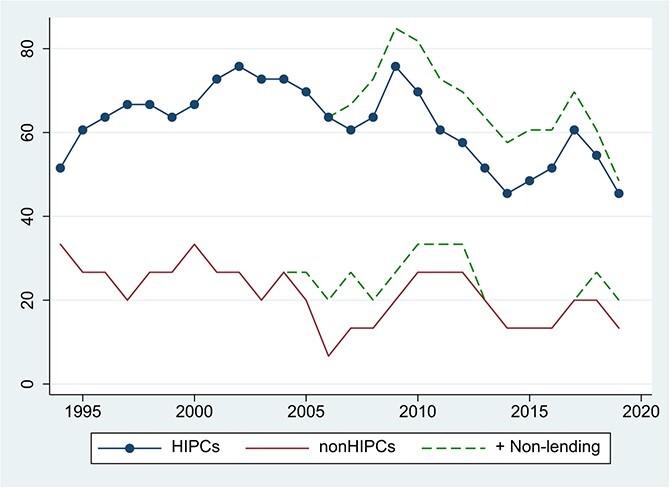
Proportion of Countries in SSA With IMF Programs. Source: IMF. Notes: The solid lines show lending programs.

Fiscal policy, in turn, continued to be closely monitored following the receipt of MDRI relief, via the annual debt-sustainability exercises required of all PRGT-eligible countries. New borrowing from multilaterals to fund poverty reduction strategies was subject to prudential assessment based on CPIA scores. The recipients of official debt relief were also subject to the World Bank’s non-concessional borrowing policy. These arrangements provided close surveillance not only of debt sustainability but also of the macroeconomic policy environment. Finally, as indicated in Figure 11, a high proportion of HIPC countries maintained IMF lending programs, which incorporated debt limits tied to the LIC DSF ratings. After 2005, these included Policy Support Instruments, voluntary monitoring arrangements without a lending component. Post-MDRI patterns therefore emerged in a period of high surveillance, in which many African governments had good reasons to exploit fiscal space via higher primary deficits financed by borrowing. Except where CPIA scores and debt sustainability ratings deteriorated sharply, they were typically able to do so with the consent and even encouragement of the multilateral institutions.

Low-income countries in SSA began to enter international bond markets beginning with Cote d’Ivoire in 2006, a full 3 years before that country received HIPC debt-service relief and more than 6 years in advance of its HIPC/MDRI debt-stock cancellation (see Figure 7). To gain access to international capital markets, countries had to be rated by private credit-rating agencies. In principle, these agencies act as complementary agencies of restraint to IMF and World Bank surveillance, underpinning macroeconomic discipline and restraining Easterly-style dynamics in the borrowing countries. Given their reliance on LIC DSF data, it is unclear whether the activities of the private rating agencies modify or mainly amplify the LIC DSF ratings. At the very least, the prominence of the rating function in sovereign bond markets underscores the potential leverage of the LIC DSF ratings over lender behavior. Among emerging-market countries, the IMF does not publish summary debt-sustainability ratings as a matter of policy, so as to avoid exacerbating the volatility of spreads and market access among the borrowing countries. We return to this issue in a later section.

### 3.4 Country experience

Our broad overview hides considerable diversity at the country level. Table 4 illustrates this point by showing some of the structural correlates of debt vulnerability in SSA in the period since 2013. State fragility and commodity-exporter status are both strongly associated with higher average levels of risk across the 2 years. Fuel exporter status is correlated with greater risk, but not when also controlling for commodity-exporter status. The CFA countries and the members of the East African Community have substantially lower average risk of distress.

**Table 4:** Dependent Variable: Average LIC DSF Distress Rating, 2013 and 2019

Country category	(1)	(2)	(3)	(4)	(5)	(6)
Fragile state	0.724^***^					0.456^**^
	(0.227)					(0.199)
Commodity exporter		0.741^***^				0.520^**^
		(0.230)				(0.226)
Fuel exporter			0.583^***^			0.253
			(0.197)			(0.247)
East African community				−0.806^**^		−0.625^**^
				(−0.363)		(−0.258)
CFA country					−0.378	−0.638^***^
					(−0.226)	(−0.178)
Constant	1.867^***^	1.917^***^	2.250^***^	2.406^***^	2.420^***^	2.030^***^
	(0.157)	(0.163)	(0.139)	(0.131)	(0.176)	(0.169)
N. of obs.	37	37	37	37	37	37
R-squared	0.21	0.228	0.042	0.126	0.052	0.497
Mean of dep var	2.297	2.297	2.297	2.297	2.297	2.297

Robust t-statistics are in parentheses (^*^, ^**^ and ^***^ denote significance at the 10%, 5% and 1% levels). Source: Risk ratings are from LIC DSAs, and country categories from Table 2. The East African Community variable excludes South Sudan, which did not accede until 2016.

The same structural correlates have considerably less traction over the deterioration in risk ratings between 2013 and 2019, as shown in Table 5. Table 5 brings in two additional variables: the number of years between a country’s receipt of MDRI relief and 2019, and the estimated annual size of Chinese official loan-like inflows to the country (as a share of the country’s GDP) between 2000 and 2012. We include the latter variable to accommodate the possibility that surveillance of these flows is imperfect even within the LIC DSF. Data on gross Chinese flows come from the project-level dataset constructed by Dreher *et al.* (2017), who introduce a methodology for using open-source information to track underreported financial flows.

**Table 5:** LIC DSF Ratings Among the African HIPCs, 2013 and 2019

Variable	Average risk rating 2013 and 2019	Increase in risk rating (0/1 dummy)	Increase in risk rating (absolute)	Average risk rating 2013 and 2019	Increase in risk rating (0/1 dummy)	Increase in risk rating (absolute)
	(1)	(2)	(3)	(4)	(5)	(6)
Time since MDRI	−0.079^**^	0.057^**^	0.084^*^	−0.011	0.103^**^	0.224^***^
	(−0.032)	(0.025)	(0.048)	(−0.063)	(0.037)	(0.074)
Chinese flows 2000–12	0.280	0.445^***^	1.135^***^	0.102	0.347^**^	0.848^***^
	(0.199)	(0.142)	(0.346)	(0.220)	(0.161)	(0.251)
Fragile state				0.351	0.215	0.467
				(0.259)	(0.183)	(0.341)
Commodity exporter				0.330	−0.266^*^	−0.364
				(0.223)	(−0.143)	(−0.279)
Fuel exporter				0.415	0.471	1.515^**^
				(0.502)	(0.320)	(0.638)
East African community				−0.477	−0.690^***^	−0.977^*^
				(−0.438)	(−0.200)	(−0.497)
CFA country				−0.419^*^	−0.307	−0.200
				(−0.230)	(−0.183)	(−0.296)
Constant	2.882^***^	−0.384	−0.898^*^	2.031^***^	−0.661	−2.310^**^
	(0.330)	(−0.244)	(−0.503)	(0.692)	(−0.482)	(−1.013)
N. of obs.	30	30	30	30	30	30
R-squared	0.135	0.301	0.391	0.407	0.600	0.604
Mean of dep var	2.133	0.467	0.600	2.133	0.467	0.600

Robust t-statistics are in parentheses (^*^, ^**^ and ^***^ denote significance at the 10%, 5% and 1% levels).

*Note:* These regressions omit Cabo Verde, Kenya and Lesotho because they were not eligible for MDRI relief. They omit Eritrea, Sudan and Zimbabwe because these countries were already in distress in 2013. They omit South Sudan because data on Chinese loan-like flows are unavailable before that country’s independence in 2012.

Time after the MDRI is positively correlated with a deterioration in risk rating between 2013 and 2019. This result is not surprising given the borrowing space created by MDRI relief. Chinese flows preceding the sample period are also strongly predictive of the subsequent deterioration in ratings, and also of the average level in 2013 and 2019. We find that broad measures of the concessionality of debt as of 2012, by contrast, have no predictive power for either the level or the deterioration in LIC DSF ratings.^14^

There is ample room for research to understand the debt-sustainability trajectories of individual countries and assess the relevance of Easterly-style concerns about over-borrowing. At one extreme, Tanzania and Uganda have maintained low-risk status throughout the post-relief period, while Rwanda moved from moderate to low in 2014. In these countries, debt relief was not dissipated in a private or public consumption boom; instead, investment to GDP ratios rose sharply between 2005 and 2013, accompanied by increase in both domestic and foreign savings. Ethiopia and Zambia seem more obvious candidates, going from low to high risk of debt distress between 2013 and 2019. But neither country shows clear evidence of Easterly-style dynamics. Ethiopia has displayed consistently high rates of investment-driven growth since 2005, while Zambia posted growth rates averaging above 5% between 2005 and 2018. Both countries had Bank and Fund surveillance under their respective programs and were subject to regular debt-sustainability assessments. Their reclassifications within the LIC DSF also occurred long after their HIPC completion points of 2004 and 2005 and receipt of MDRI relief in 2006 (a result consistent with Table 5). The viability of debt-financed public investment projects is of course a crucial issue in these countries, as it is throughout the region. Any portion of public investment that is unproductive in *ex ante* terms is the equivalent of debt-financed consumption in th Easterly framework and may point to similar political-economy drivers of over-borrowing.

Ghana’s debt-sustainability deterioration has clearer Easterly-style elements, along with a major role for exogenous shocks. The scope for inexpensive borrowing expanded in Ghana starting in the mid-2000s, reflecting a combination of debt relief, steady economic growth since the mid-1980s, low global interest rates and the emergence of oil-export prospects. Against a background of fiscal dominance, the adoption of an inflation-targeting regime in 2007 may have contributed to market expectations of enhanced fiscal discipline. Beginning in 2012, however, the government financed large increases in the public-sector wage bill through both external and domestic debt. Borrowing costs then skyrocketed as external debts mounted and global oil prices collapsed starting in 2014. A risky fiscal path rapidly became unsustainable. Market responses accelerated the process, by driving up interest rates on the non-concessional portion of debt and exacerbating the real depreciation associated with falling oil prospects.

### 3.5 Summing up

The HIPC/MDRI period was part of a longer episode that began in the early 1990s and combined major and sustained policy reforms with a revival of growth. Reforms are absent in Easterly’s framework by design, but they can readily be incorporated. The growth diagnostics framework of Hausmann *et al.* (2005), for example, adopts a similar endogenous growth formulation but breaks Easterly’s assumed equivalences between private and social returns to investment, domestic and global interest rates and (especially) the return to investment and the cost of external borrowing. Along a balanced-growth path in that model, consumption and the private capital stock grow at the following rate (cf. equation (3)):

(4) \begin{equation*} \frac{{\dot{C}}_t}{C_t}=\frac{{\dot{K}}_{PRI,t}}{K_{PRI,t}}=\sigma \cdot \left[\left(1-\tau \right)\cdot{A}^S\left({A}_t,{H}_t,{K}_{PUB,t}\right)-r\left({r}_t^{\ast },\rho \right)\right], \end{equation*}

where the first term in square brackets captures influences on the return to investment and the second captures the cost of finance. In that framework, private investment and growth respond to the return to investment as in (3), but also to reductions in distortionary implicit or explicit taxes on private investment ($\tau,$ potentially influenced by debt overhang effects); increases in human capital ${H}_t$ and/or public infrastructure capital ${K}_{PUB,t}$; and reductions in the cost of domestic and/or external finance, with the former proxied by the same $\rho$ that appears in Easterly’s model and the latter by ${r}_t^{\ast },$ which would reflect global interest rates as well as debt sustainability concerns. As we will see in Section 5, Buffie *et al.* (2012) develop a fully-articulated general-equilibrium model that incorporates all of these features, along with diminishing returns to capital.

In a contest of parables, Easterly’s is more elegant but the HRV and Buffie *et al.* frameworks incorporate a set of development roles for borrowing and capture many avenues for growth-promoting reform that were pursued in SSA both in advance of the HIPC/MDRI era and during it. The central story of the period since the late 1980s, as we see it, is one of durable policy reforms that transformed the investment and growth environment throughout SSA. Multilateral debt relief appears to have played an important role in facilitating these reforms, through a combination of policy conditionality and monitoring, outright resource transfers and improved scope for new borrowing. Africa’s development debts emerged under the scrutiny of a system that was designed from the outset to balance concerns about over-borrowing and over-lending against the need for external resources to support development goals. Doing justice how this tension evolved at the country level requires an approach in which Easterly-style political-economy considerations operate alongside realistic normative motivations for debt accumulation.

## 4. Proximate drivers of African indebtedness, 2006–2018

In this section we highlight the proximate drivers of African indebtedness since MDRI, focusing on changes in the debt to export ratio and debt to GDP ratios. We first document the role of global commodity export markets in shaping debt accumulation episodes in the region. Next, we identify and assess the relative influence of key drivers of changes in indebtedness, by using equation (2) to do an accounting-based decomposition of changes in the debt to GDP ratio. We end by taking up the issue of valuation effects associated with exchange rate movements.

### 4.1 The debt-to-export ratio and the terms of trade

**Figure 12:**
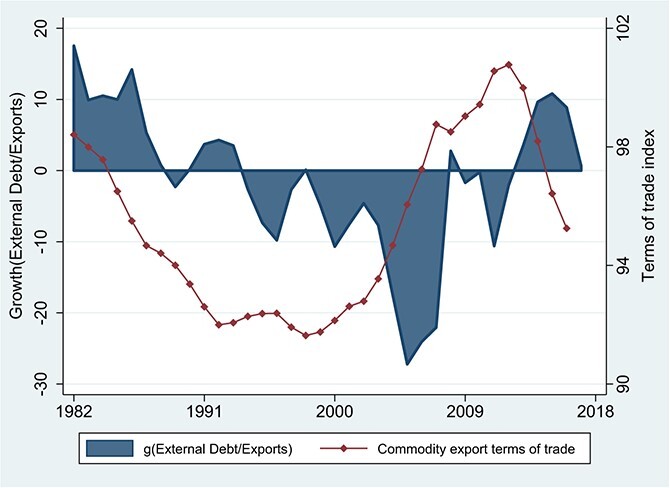
SSA: The External Debt/Export Ratio and Commodity Export Terms of Trade. Source: External debt and exports in U.S. dollars are from the World Bank, World Development Indicators online. The commodity export terms of trade is from Gruss and Kebhaj (2019). Note: The figure is based on 3-year centered moving averages of estimated time effects in a panel regressions with unbalanced samples. The samples for debt and exports include all country/years for which both nominal dollar values are available. The terms of trade sample includes all country/years in the debt/export sample for which the commodity terms of trade is also available.

Persistently weak growth in the dollar value of exports played a crucial role in the accumulation of external debt and the emergence of debt-servicing problems in Africa during the 1980s (Humphreys and Underwood, 1989). Figure 12 brings that earlier period into view and emphasises the destabilising impact of global commodity price movements on Africa’s debt dynamics.

The blue shaded areas in Figure 12 give the difference between the growth rates of nominal debt and nominal exports in US dollars. The debt-to-export ratio rises when this difference is positive and falls when it is negative. Three distinct phases emerge. The first runs from the early 1980s to the mid-1990s and is a phase of economic stagnation and mounting debt problems as the growth of debt outpaces that of exports (mirroring the trend we noted earlier in the debt-to-GDP ratio). Internal and external drivers were both influential during this phase, which combined anti-growth policy regimes with slow global growth and a protracted decline in commodity export prices. The second phase is the debt cancellation/policy-reform episode, which runs from the mid-1990s to the onset of the global financial crisis in 2008. During this phase, the debt-to-export ratio is falling through a combination of debt cancellation and strong growth in export revenues, with a long boom in the primary commodity terms of trade playing an important role. The third and current phase is one of debt accumulation, driven in part by favorable borrowing conditions and initially matched with robust growth in exports. As with the debt-to-GDP ratio, the debt-to-export ratio begins to rise on average for the region only after 2012, a development closely timed to the sharp reversal in commodity export prices starting in 2013.

### 4.2 Proximate drivers of the debt-to-GDP ratio

**Figure 13:**
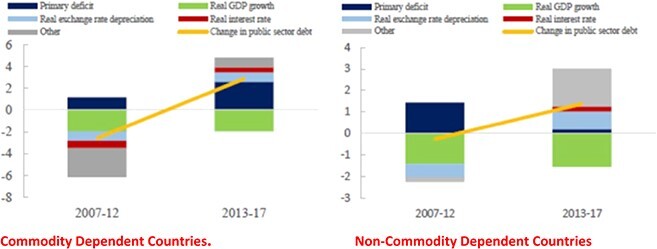
Decomposition of Sources of Growth of Indebtedness—IDA Countries. Source: International Development Association (IDA) (2018), Figure B2.1, p. 6.

Figure 13 divides the post-MDRI period into two sub-periods and illustrates the use of equation (2) to identify the proximate drivers of observed changes in the debt to GDP ratio. Noting that the real interest rate measured in domestic goods, ${r}_t={i}_t+{\hat{e}}_t-{\pi}_t,$ can be written as the sum of a real interest rate measured in foreign goods and the rate of real depreciation of the local currency $({r}_t={r}_t^{\ast }+{\hat{rer}}_t),$ equation (2) implies that changes in the ratio of public debt to GDP can be decomposed into the contributions of the primary deficit, real exchange rate depreciation, real GDP growth, the real interest rate and a residual. The sample in Figure 12 comprises all IDA-eligible countries, the majority of which are in SSA.

Comparing 2013–17 with 2007–12, the dynamics switch from a falling to a rising debt ratio, with a much larger increase among commodity-exporting countries. For the commodity-exporting group, the contributions of the primary deficit and real interest rate are significantly enhanced after 2013, consistent with a slow public-expenditure response to collapsing commodity-based fiscal revenues and with a hardening of borrowing terms. The calculation shows a substantial contribution from real exchange rate depreciation, reversing the smaller but favorable contribution in the earlier period. Among non-commodity exporters, the effects of real GDP growth and real exchange rate depreciation are dominant, while the primary deficit moves virtually into balance on average.

Consistent with Figure 13, we briefly stress three main drivers of the recent surge in indebtedness in SSA: greater ability to borrow, increased financing needs and fragility. The relative influence of these factors varies significantly across time and countries (Christensen and Schanz, 2018). First, the borrowing space African countries were able to exploit resulted from a combination of the reforms and debt-stock relief reviewed in Section 3 and favorable global financial conditions. By removing the overhang of unsustainable debts, the HIPC Initiative made it possible for countries to exploit the borrowing capacity generated by faster growth, a process that was underway in response to economic reforms in many countries well before the HIPC Decision Point was reached. The MDRI then went further, cutting debt levels to far below sustainability thresholds in order to enhance the fiscal space for meeting the MDGs.

The LIC DSF assessments that were integral to the post-2005 lending and surveillance activities of World Bank and IMF also played an important informational role by certifying improvements in borrowing capacity and reducing the cost of screening for private and bilateral official creditors including China. Finally, access to relatively inexpensive borrowing improved for exogenous reasons both during and after debt relief. These include the long boom in commodity export prices shown in Figure 12, as well as interest-rate reductions that occurred across the maturity spectrum as advanced-country central banks adopted quantitative easing in response to the global financial crisis. Brauning and Ivashina (2018) find that changes in US bond rates feed through more than point-for-point to emerging-market sovereign bond rates, a phenomenon observed both during and after the global financial crisis as global investors searched for yield.

Second, Africa’s increased financing needs reflect in part the drives to fill the region’s huge infrastructure gap (Ndulu *et al.*, 2007, 2008) and to achieve the ambitious MDG targets, particularly those related to human capital and welfare. External financing also played a countercyclical role in the face of the global growth slowdown and terms of trade shocks that not only weighed on growth and government revenue but also led to a rise in government contingent liabilities (Christensen and Schantz, 2018; Christensen, 2016).

Finally, debt accumulation reflects the fragility of a number of economies in terms of civil strife, susceptibility to natural disasters and dependency on volatile commodity markets. Consistent with Table 4, the World Bank reports that all small island economies, more than 90% of fragile countries, and more than 85% of commodity exporters, are classified either at high or moderate risk of debt distress (IDA, 2018).^15^ Since these factors are largely exogenous, depending on their weight in influencing debt risks faced by the country, they may dampen the impact of policy-based actions for debt management.

### 4.3 Implications of exchange rate flexibility

When external debt is denominated in foreign currency, exchange rate movements can have a large impact on the burden of debt as measured in domestic currency. Figure 13 illustrates this effect for African HIPCs with independent national currencies, nearly all of which made a transition to greater exchange rate flexibility over the course of the 1990s. We show the external debt to GDP ratio ${d}_t\equiv{e}_t{D}_t/{P}_t{Y}_t$, along with a version of that ratio that holds the real exchange rate ${e}_t/{P}_t$ constant and therefore isolates movements in the ratio of US dollar external debt to constant-local-currency GDP ($D/Y$). This separation of the debt ratio into ${e}_t/{P}_t$ and ${D}_t/{Y}_t$ is highly artificial; the two components are jointly determined, and also movements between the dollar and other currencies of denomination can exert sizeable valuation effects on the external debt measured in dollars. But with appropriate caveats we can say that conditional on the path of ${D}_t/{Y}_t,$ the long decline in HIPC debt-to-GDP ratios was strongly reinforced, during most of the 2000s by a long-term real appreciation against the US dollar.

Medium-term real appreciation is of course an equilibrium response to a favorable combination of terms of trade movements and capital inflows, as it is to long-term economic growth via the Harrod–Balassa–Samuelson effect. Figure 1 is therefore consistent with a role of the real exchange rate in lending a procyclical component to creditworthiness in countries that borrow mainly in foreign currencies. In favorable periods, this effect operates via the influence of real appreciation in pushing the debt to GDP ratio down further (and similarly for ratios of debt or debt service to fiscal revenue, other things equal). In the background, this link between the terms of trade, financial inflows and the real exchange rate operates to stabilise aggregate demand and provides one of the leading arguments for flexible exchange rates among countries with volatile export proceeds. But it also exerts a procyclical impact on the debt burden among countries that borrow in foreign currency.

At higher frequencies, the stickiness of domestic prices means that movements in the nominal exchange rate tend to pass through substantially to the real exchange rate. Nominal exchange rate movements can therefore exert large valuation effects on the debt ratio. As indicated in Table 6, real exchange rates are highly volatile among low-income countries, and particularly among those with flexible nominal exchange rates. Among African HIPCs, the real exchange rates is more than twice as volatile at all horizons among countries whose currencies are not irrevocably pegged to an advanced-country currency or basket—i.e., outside of the CFA zone—as they are in the CFA zone. When debt is held by private creditors this can generate a highly procyclical channel for market perceptions in any country not operating on a hard peg. In this scenario, creditworthiness concerns lead to nominal and real depreciation, which immediately worsens the debt burden and increases the country’s exposure to rollover risk. This is an area in which empirical research can help low-income countries with increased exposure to cross-border commercial debt flows understand the sources and consequences of exchange-rate volatility and their interaction with debt vulnerabilities.

**Table 6:** Volatility of Within-Country Cumulative Percentage Change in REER, 2001–2017

Country group	n			Horizon (years ahead)		
	1	2	3	4	5	6
SSA HIPCs	31	6.9	10.4	12.4	13.6	14.3	15.6
CFA	12	3.8	5.7	6.8	7.0	7.2	8.1
Non-CFA	19	8.8	13.3	15.9	17.8	18.8	20.3
LICs	27	7.5	11.3	13.2	14.4	15.4	16.7
LMICs	36	5.7	8.8	10.9	12.9	14.4	15.5
UMICs	39	5.8	8.7	10.6	12.2	13.6	14.9
HICs	53	4.6	7.2	8.8	10.1	11.1	11.9

*Source:* CPI-based annual trade-weighted real effective exchange rate indexes calculated with 172 trading partners, as described in Darvas (2012) and http://bruegel.org/publications/datasets/real-effective-exchange-rates-for-178-countries-a-new-database/

*Note:* The table shows simple averages across the country group of within-country standard deviations of the k-year-ahead cumulative appreciation of the real effective exchange rate index (2007 = 100). Countries with an REER index below 33 or above 300 for any year are excluded from the table. LICs, LMICs, UMICs and HICs are countries classified by the World Bank as low-, lower-middle, upper-middle and high-income in 2017.

## 5. Development assets at risk

Buffie *et al.* (2012) develop an approach to debt management that embeds the debt-sustainability accounting in Section 2 in a dynamic open-economy general equilibrium model. This approach incorporates forward-looking consumption behavior as in Easterly (2002) but also provides a fully articulated treatment of major elements of the Hausmann *et al*. (2005) framework we referred to earlier. The framework has been used to explore debt-management issues in a number of African countries, particularly in the context of surges in infrastructure investment and elevated levels of non-concessional debt.^16^ We use it here to extend our discussion of debt sustainability and introduce the concept of development assets at risk.

Private firms in the Buffie *et al.* analysis produce (traded and nontraded) output by combining capital with labor and the services of public infrastructure capital. Households own the domestic capital stock and the stock of domestic government bonds and can borrow abroad at a premium above the rate paid on non-concessional foreign debt by the government. The government collects a value-added tax and user fees on public infrastructure services and combines these sources of revenue with domestic and foreign borrowing to finance expenditures on debt service, transfers to the household sector, maintenance of public infrastructure and investment in new infrastructure. The quality of public infrastructure services can vary, as can the efficiency with which the public sector transforms investment expenditures into productive infrastructure. For given parameters, however, capital is subject to diminishing returns. *Ceteris paribus*, a lower capital stock implies a higher marginal product of capital.

The model builds in all of the major normative motivations for borrowing in the context of a low-income country, including a high marginal product of public and/or private capital, a desire to smooth public and/or private consumption over time, and a low cost of concessional or (at least episodically) non-concessional borrowing. For given fiscal settings including the path of public investment, debt sustainability requires that the government maintain continuous debt service while ultimately achieving a primary surplus sufficient to stabilise its debt to GDP ratio. Concessional borrowing is assumed to be available on given terms and in predetermined supply (as in Easterly, 1999, 2002), so the analysis focuses on stabilising non-concessional claims.

Debt distress in the Buffie *et al.* analysis is tied to limits on fiscal adjustment. Debt is sustainable if a government that borrows to finance an infrastructure push can repay the resulting debts without raising taxes to intolerable levels or imposing politically unviable reductions in transfers to the household sector (the latter stands in for many forms of current spending including social spending and public sector wages). A government that cannot adjust its primary deficit rapidly enough and/or by a sufficiently large amount faces explosive debt dynamics, as in Buiter (1985) and the LIC DSF. Exogenous threats to sustainability include increases in global interest rates and/or country risk premia, deteriorations in the terms of trade and natural disasters. Endogenous threats include poorly-chosen or badly-implemented projects, inadequate provision for recurrent costs and over-reliance on domestic financing (given its high *ex ante* cost as compared to non-concessional external financing).

Among these threats, the mismanagement of recurrent costs may have played an important role in reducing the growth impacts of investments in physical and human infrastructure in the pre-HIPC period. Analyses of infrastructure projects in Africa have shown that the projected economic rate of return on these projects is generally high; Briceno-Garmendia *et al.* (2004), for example, report an average anticipated return of 35% for World Bank infrastructure projects implemented between 1998/99 and 2002/2003.^17^ But inadequate operation and maintenance provisions in recurrent budgets can sharply reduce the *ex-post* returns to infrastructure investments (Adam and Bevan, 2015). Given the size of recurrent costs, key drivers of the recurrent cost problem relate to the limited appropriability of economic returns. The key elements are present in the Buffie *et al.* (2012) model, including difficulties in raising appropriate user charges due to regulatory forbearance and political expediency, along with limits on distortionary taxation (Adam *et al.*, 2017).

Recurrent cost pressures may be intrinsic to some degree to the differential constraints low-income countries face in financing current and capital spending. In low-income African countries, development budgets typically face a softer budget constraint, given the availability of official and private external financing to supplement any contribution from the country’s recurrent surplus. Recurrent expenditures, in turn, are predominantly financed by domestic revenue, with wages and debt service comprising the bulk of expenditures and taking near-absolute priority. Operation and maintenance expenditures therefore become a residual. The pace of non-fungible external project finance can therefore run ahead of the growth of domestic revenue available to maintain and operate these assets. This mismatch worsens endogenously when external shocks or fiscal indiscipline generates adverse debt dynamics and produce large increases in debt service, raising the risk of asset deterioration or inability to generate the services from them.

A crucial element of the Buffie *et al.* (2012) analysis is the strong long-run crowding-in effect of public infrastructure investment on private investment for standard parameterisations of the model. What the analysis stresses, however, is that even if public infrastructure investment is fully self-financing in the long run through a combination of user fees and growth effects, the ability to exploit the lower expected cost of non-concessional external borrowing to cover a portion of initial investment costs can be crucial to the fiscal viability of the project. This rationale for non-concessional foreign borrowing combines the investment-productivity and consumption-smoothing arguments for external borrowing with the public-good aspects of public infrastructure. In the absence of external borrowing, the up-front costs of an infrastructure push (net of fixed concessional financing) create a fiscal gap that has to be financed by a combination of domestic borrowing and fiscal austerity. These responses crowd out private investment through higher real interest rates, and consumption through smaller transfers, slower growth and a higher VAT rate. The result is a weak or negative revenue response that may require an intolerable degree of austerity even on the transition path to a fiscally sustainable outcome. By providing outside resources, external borrowing can soften the up-front costs in terms of fiscal adjustment, private investment, consumption and growth, of financing a public infrastructure push. Buffie *et al.* (2012) show that exploiting the scope for non-concessional borrowing can generate significant welfare improvements and even render an otherwise infeasible investment plan feasible—while also, of course, exposing the borrower to debt sustainability risks.

Among its other implications, this analysis suggests a simple rejoinder to the Easterly argument about HIPC debt relief: in the presence of productive public investment opportunities, a debt cancellation that simply makes room for new external borrowing—even on non-concessional terms—can generate important gains in welfare.

We have emphasised the dangers of under-provision for recurrent costs, particularly as debt dynamics deteriorate. A similar argument may apply to the infrastructure projects themselves as the risk of debt distress increases. This element is not present in the Buffie *et al.* (2012) analysis, which treats the public infrastructure budget as sacrosanct even on dynamic paths that violate debt-sustainability constraints. But in the absence of bond covenants or other restraints that limit sovereign control over these projects, it is not clear from a positive perspective what protects the public investment budget from the fiscal pressures associated with an elevated debt burden. Quite the contrary, in fact: unlike higher VAT rates, lower public fuel subsidies and reduced public employment, all of which are subject to immediate sociopolitical constraints, an interruption of public investment may not have much better protection than the deferral of necessary operations and maintenance expenditures. This of course was Easterly’s concern, but we are reviving it here in a very different context: in a situation of debt pressure, what protects development assets that are at risk from the behavioral responses of borrower governments? Broadening the question further, what protects these assets from the responses of creditors, who also hold direct or indirect claims on these assets? The latter question is the central preoccupation of domestic bankruptcy laws and lies at the heart of contemporary efforts to develop institutions for orderly workouts of sovereign debt.

We will return to a discussion of debt workouts in a later section. But the logic of the Buffie *et al.* model can be extended to human capital, and in our view, this is an important extension given the productive role of human capital and the importance of public education and health expenditure in human capital formation. Such an extension would immediately widen the concept of development assets at risk. HIPC conditionality centered on achieving macroeconomic stability, as a requirement to qualify for debt service relief, and then on the development and implementation of a national Poverty Reduction Strategy, as a condition for receiving irrevocable debt stock relief. The latter element can be viewed as a straightforward *quid pro quo*, required to convince donor-country constituencies to fund the Initiative. But it is also consistent with the view that debt service requirements were crowding out essential social spending. The latter view was later made explicit in the move to the MDRI, which was designed to crowd in spending on the MDGs.

There are many reasons why it may be more appropriate to finance even highly productive social spending through domestic tax revenues and grants rather than through user fees and non-concessional debt. These include the discouraging effects of user fees on demand, the long gestation lags for the relevant economic returns (the relevant lags may be partly inter-generational, in the case of education and health investments in girls) and the fact that human capital investments are embodied in individuals and cannot be collateralised. Human assets are nonetheless at risk during periods of macroeconomic stress, as fiscal austerity undermines public and private expenditures on health and education. Given the dual status of education and health as merit goods and productive assets, and the highly unequal distribution of these assets both within and across countries, there are important equity and efficiency arguments for protecting social spending during fiscal austerity. Maternal and child health services, for example, cannot be deferred without being lost completely, with long-lasting effects on maternal health and child cognitive development that are likely to be concentrated among the poorest. From a normative perspective, how should these costs in human capital formation be balanced against the costs of delaying the completion or maintenance of public infrastructure capital? From a positive perspective, what forms of spending or public assets are most likely to be protected in a situation of debt distress?

As this discussion suggests, incorporating human capital into a debt-sustainability framework will introduce normative and positive dimensions that may strongly influence debt-management choices and their consequences. Atolia *et al.* (2017) make a start on the positive side, by examining the balance between economic infrastructure (ports and roads) and social infrastructure (schools). Their hypothesis is that low-income countries under-invest in social infrastructure because the scope for patronage is greater on economic infrastructure projects. There are important avenues for further research here, including attempts to distinguish patronage-driven patterns from other drivers—for example, the emergence of China as a major new source of official funding for hard infrastructure, and/or the non-fungibility of non-concessional finance for infrastructure projects—that may produce a similar pattern of public investment.

Research on the composition of public infrastructure spending should also address important normative questions. Growth theories in which human and physical capital are complementary inputs into production tend to imply that there is an optimal balance between the two and that deviations from the optimal balance effectively convert one form of capital into a binding constraint on achievable growth. Deviations from the appropriate balance can therefore imply sharp divergences in optimal investment rates along a transition path that establishes the optimal balance (Acemoglu, 2009). Do national balance sheets on a country by country basis currently reflect a broadly appropriate balance of past public spending on economic and social infrastructure? And, what should the balance of public investment effort be looking ahead?

**Figure 14:**
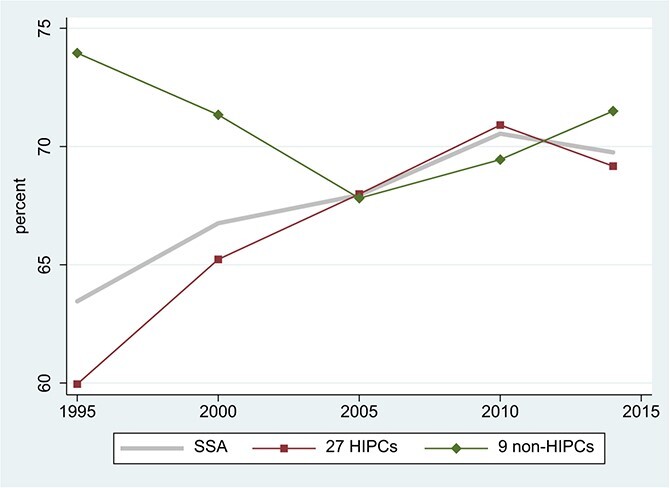
SSA: Share of Human Capital in Accumulable Capital. Source: World Bank, Wealth of Nations database online. The Figure shows averages across the 36 countries in SSA with available data for all periods.

Figure 14 uses the World Bank’s new Wealth of Nations database to track the ratio of human to physical capital (private plus public) in SSA, comparing cross-country averages for HIPC and non-HIPC countries at the 5-year intervals of the data. The ratio differs sharply between the two groups, showing a sharp increase in the relative weight of human capital among the HIPCs, in both absolute and relative terms. This pattern is consistent with the strong emphasis on social expenditures during the MDG and HIPC/MDRI campaigns. Its implications for the current conjuncture, however, remain unclear. If human capital investment represented a binding growth constraint among HIPC countries over the early part of the 2000s, these data suggest a pivot towards balance over time, and possibly a situation in which the environment for public and private physical capital accumulation now represents the more binding constraint. There is ample scope for research that incorporates human capital into a Buffie *et al.*-style framework and uses microeconomic evidence to tie down key parameters that govern the returns to alternative forms of public spending. Such work can help inform difficult fiscal choices in a period of rising debt.

A final development asset is the commitment to market-supporting policies, including strong macroeconomic fundamentals and improved economic governance, which began to take root in the mid-1990s and was documented in the African Economic Research Consortium (AERC)’s Growth Project (Ndulu *et al.*, 2008) and elsewhere. On the macroeconomic management side, debt pressures will test these accomplishments by stretching the adjustment capability of fiscal institutions and the ability of central banks to manage market volatility and maintain an anchor for inflation. Improved debt-management capabilities will be crucial for maintaining an effective relationship between these institutions and avoiding a shift back towards fiscal dominance.

## 6. New issues of creditor coordination

Table 7 draws on IMF data to provide further compositional details on the changing structure of public and publicly guaranteed debt in low-income countries. The table compares 2007 and 2016 and contrasts the experience of eight post-HIPC countries in SSA who were either at high risk in 2018 or in distress, with that of the full sample of 37 low-income countries. Two main observations stand out. One is that despite the increasing profile of domestic debt in both groups over time, developments in external rather than domestic debt are what distinguish the two sides of the table. The second is the remarkable compositional shift of external debt towards new external creditors in both groups. The Paris Club bilateral creditors are virtually absent by 2016: their share of external debt falls by 71% in the full sample and by almost 80% in the SSA HIPCs with debt-distress pressures. Multilateral creditors (essentially the residual category) hold a stable share in the full group and increase their share from 20% to only 24% in the debt-pressures group. Meanwhile, China’s share of external obligations, negligible in these data at the outset of the period, rises to 11% in the overall sample and to twice that—22%—in the debt-pressure subsample. The claims of commercial creditors rise by the same amount as China’s, from a higher base. Taken together, commercial creditors and China held an average of 26% of all external public-sector debt in the low-income country sample in 2016, and the outright majority of claims—51%—in the eight SSA HIPCs with debt-distress pressures.

**Table 7:** Composition of PPG Debt in Low-Income Developing Countries, 2007 and 2016

Total PPG debt by creditor, 2007 and 2016 (% GDP)	All 37 LIDCs with data		8 SSA post-HIPC LIDCs in debt difficulties	
	2007	2016	2007	2016
**Total**	**47.1**	**52.7**	**44.7**	**72.1**
**External**, o/w	**36.5**	**37.3**	**32.8**	**53.4**
Bilateral Paris Club	7.4	2.2	8.0	2.8
Bilateral China	0.3	4.2	0.2	11.6
Commercial	2.7	5.6	4.9	15.3
**Domestic**, o/w	**10.5**	**15.3**	**12.0**	**18.7**
Marketable	3.1	7.0	5.9	9.1
Nonmarketable	7.4	8.3	6.1	9.6

*Source:* IMF (2018a).

*Note:* LIDC refers to low income developing countries. Of the 59 such countries (in all regions), 37 had continuously available data between 2007 and 2016. The 8 HIPC countries in the right-hand panel were either at high risk of debt distress or in distress in 2016. They are Cameroon, Chad, Congo, Ethiopia, Ghana, Mauritania, Mozambique and Zambia.

The engagement of SSA’s HIPCs with international sovereign bond markets started with Cote d’Ivoire in 2006, as discussed earlier. The exit of the Paris Club bilateral creditors accompanied the HIPC/MDRI process and remained in place through a deliberate shift toward grants. Chinese official flows started from low levels in the early 2000s and grew rapidly. China—not a member of the Paris Club—inaugurated its infrastructure-focused *Forum for China-Africa Cooperation* in 2006, the year the multilaterals implemented the MDRI by granting deep debt-stock relief to all countries that had reached the Enhanced HIPC completion point. Africa’s pivot towards commercial borrowing implies, as we have stressed, that the external position of African countries is more exposed to changes in global interest rates and financial market sentiment than it was even one decade ago. The entry of new creditors, in turn, implies that new issues of creditor coordination will have to be navigated in order to secure protective debt restructurings and handle payments crises regardless of their origin. We focus briefly here on the second of these issues.

Guzman *et al.* (2016) and Stiglitz and Heymann (2014) survey an extensive literature on creditor coordination in sovereign debt restructurings. Given the increasing profile of private creditors lenders in Africa, the lack of effective mechanisms to secure the constructive participation of private creditors remains extremely important, despite recent global progress in clarifying creditor seniority in sovereign bond markets and narrowing the contractual option of creditors to hold out when a debt restructuring is needed (similar developments have not occurred for bank lending). We focus here, however, on two issues related to the coordinating roles of official creditors.

First, as commercial debt increases in importance in low-income countries, how do the multilaterals navigate their simultaneous roles as lenders, credit raters and organisers of collective action among creditors? The Paris Club states that its members ‘are committed to using the IMF and World Bank Debt Sustainability Framework (DSF) for low-income countries as a reference to inform their lending decisions in light of debt distress risk assessments’ (www.clubdeparis.org). The DSAs are therefore meant to discourage over-borrowing by governments from both the demand side—by providing early warnings of needed fiscal adjustments—and the supply side—by preventing lenders from diluting the value of existing claims and raising the risk of distress through over-lending. In the absence of an ability to compel lenders, however, the rating function of the IMF may in situations of stress exacerbate repayment problems on balance, by triggering rollover risks among private lenders. How this conflict of interest between the Fund’s rating function and its lending/debt-resolution function can be resolved is an important question.

Second, how will China’s emergence as a global power and a major lender affect the financing and policy environment for African countries facing debt stress? As emphasised by Dollar (2018), many of the African countries that have borrowed significantly from China are not in debt distress. Among those that are at high risk or in distress, China has shown willingness to restructure debts on a bilateral basis (Ethiopia’s restructuring of railway infrastructure debts is a prominent example). But the essence of the donor coordination problem is that the recipient’s capacity to repay is finite and is reduced at the margin whenever any individual creditor chooses to take payment rather than rolling over its claims. Exit or holdout by China would therefore undermine the interests of other creditors; exit or holdout by other creditors would undermine the interests of China. The possibility of an impasse among creditors that creates a liquidity crisis or protracted debt overhang cannot be discounted—and is not solved by the highly collateralised nature of China’s lending because the transfer of asset ownership in default is highly likely to create deadweight losses and/or reduce the development value of the assets, and therefore to dilute the claims of other creditors.

African countries therefore have a strong collective interest in effective cooperation between China and other creditors. There are serious difficulties in the way of achieving this, particularly in the context of renewed great power rivalry in global affairs. China has a fundamental interest in preserving its capability to operate unilaterally, as it has done to date. The Paris Club no longer commands a dominant presence as a creditor group in Africa. The IMF has the best technical capabilities and the greatest experience in debt resolution, but may struggle to retain a neutral profile given the influence of its largest shareholder. On the African (borrower) side, the desire of individual countries to retain low-cost access to both private and official finance may create free-rider problems in mounting a vigorous region-wide effort to create an effective forum for cooperation. This is an area where Africa’s largest economies and its regional organisations have an important role to play in achieving a critical mass of collective effort.

## 7. Conclusions and questions for research

By contrast with the debt crisis of the 1980s and 1990s, debt-sustainability concerns are not region-wide in SSA and the policy environment for growth remains robust in most cases. A long period of favorable borrowing conditions had nonetheless come to an end well before the COVID-19 pandemic arrived in 2020, with interest rates exceeding underlying growth rates at the margin in most countries and commercial creditors increasingly sensitive to rollover risks. Fiscal adjustments were well underway in a number of countries, with the IMF forecasting a stabilisation of regional average debt to GDP ratios at roughly 55% under largely favorable external conditions (IMF, 2018b, 2019). Debt levels nonetheless continue to reflect a decade of aggressive borrowing in many cases, and there are substantial risks looking ahead, now greatly exacerbated by the emerging economic impacts of the pandemic.

We have argued that Africa’s last debt crisis, finally resolved after two decades of bilateral and ultimately multilateral debt relief, is an imperfect model for what lies ahead. Recently, accumulated debts are not only shorter term but also predominantly from commercial sources and new official donors, and thereby subject to more volatile market interest rates and sentiments as well as to new challenges of creditor coordination. More fundamentally, there are development assets at stake after two decades of investment and growth, including hard-fought improvements in policy and institutions, major public infrastructure projects nearing fruition and cumulative increases in human capital formation that in most countries continue to await a transformative response of private investment.

One clear lesson from a decade and more of debt accumulation is that the risks associated with non-concessional debts and new creditors require countries to enhance their own debt-management capabilities, particularly as a number of low-income countries are set to graduate from IDA status. This agenda may need to be more consciously embraced in multilateral country programs, with a view to incorporating capacity-building programs that pay attention to the expansion of external market-based financing and that are capable of operating in a multi-creditor environment. The IMF’s Multi-Pronged Approach to debt management (IDA, 2019) becomes pivotal for this transition.

Looking ahead, it seems equally clear that the economic impacts of the COVID-19 pandemic will substantially complicate the adjustment process that was underway in advance of early 2020. Coulibaly *et al.* (2019) cite the reversal of key drivers—the stabilisation of commodity prices, the firming up of economic growth, reduced exchange-rate pressures and some initial fiscal consolidation efforts—as explaining the stabilisation of debt ratios in 2017 and their projected decline by 2023. The IMF noted these developments in its *Africa Regional Economic Outlook* reports for 2018 and 2019 and saw African growth rebounding from 2.7% in 2017 to 3.5% in 2019 before reaching 4% in the medium term. In forecasting a rapid stabilisation of debt ratios region-wide, the IMF also anticipated broadly accommodative external financing conditions.

Local and global responses to the pandemic will place pressure on each element of these projections, including favorable assumptions about external finance. They will also exacerbate key existing concerns, including the IMF’s observation that fiscal imbalances were in most cases being contained through a combination of higher commodity revenues and sharp cuts in capital spending, with little progress on domestic revenue mobilisation (IMF, 2018b, 2019).

Against this background, we have stressed three challenges that African borrowers must contend with in the context of elevated debt. The first concerns those countries already under stress from debt obligations that are maturing ahead of the revenue streams from the infrastructure investments (physical and social) they financed. The challenge here is to address maturity mismatches without recourse to destructive adjustment patterns that compromise ongoing projects by cutting capital budgets, starve infrastructure assets of recurrent expenditures for their maintenance and operation or simply monetise large primary deficits with the resultant macroeconomic instability. A number of countries—including Kenya, Ethiopia, Nigeria and Ghana—have managed to roll over commercial debts through restructurings that exchange lengthening maturities for modest increases in interest rates. Although these restructurings have applied mainly to Eurobonds or private placements, Ethiopia was able to restructure $4 billion in Chinese debt arising from a major railway project, lengthening the maturity of these obligations from 7 to 30 years. Pressures for restructuring will be particularly high in 2020, when there is a concentration of maturing obligations across African countries (Christensen and Schanz, 2018).

Some countries are considering refinancing local-currency debt with foreign-currency debt to take advantage of lower interest rates for the latter. Public debt denominated in domestic currency confronts the bearer with inflation risk and therefore tends to require a higher expected yield than the same debt issued in foreign currency. This opens up possibilities for reducing expected borrowing costs by swapping domestic-currency for foreign-currency claims, but this bargain can be treacherous in a situation of high exposure to distress.

The second challenge relates to the plurality of creditors, which complicates coordination in the event of resolution and may make this process much more protracted. We dealt with this issue in detail in Section 5. Issues of creditor coordination are significantly more daunting now than they were the mid-1990s, reflecting the increasing importance of private creditors and the emergence of China as a major source of official finance.

The third challenge relates to rollover risks in light of greater exposure to market-based external financing. Countries facing increased exposure to global market volatility require enhanced domestic capacity for real-time analysis of the drivers of exchange rates, global interest rates and risk premia. The context requires not only a capacity for agile policy responses but more fundamentally an enhanced capacity to understand and interact with key market players, including rating agencies.

These challenges call for new research, as we have emphasised throughout the paper. We close by outlining some of the most promising directions.

• Does the increase in current spending following MDRI observed by Cassimon *et al.* (2015) survive controlling for the variables like the terms of trade and partner growth rates, so as to allow a differential effect of the global financial crisis? If current spending did increase following debt-stock relief, what do we know about the nature of the spending? Was it impatient consumption, or was it human development spending (education and health) and in that sense both a vindication of the MDRI design and a response that was closer to investment than to consumption?

• Education and health capital are productive assets, especially in a world of rapid technological innovation. From a normative standpoint, do these forms of spending warrant special protection from fiscal austerity on growth and/or distributional grounds? Budgetary concepts of capital and current spending may be a pitfall here, if they correspond poorly to analytically relevant concepts of development assets and their maintenance. The normative issues, in turn, may require working with extensions of Buffie *et al.* (2012) that incorporate human capital. From a positive standpoint, what categories of public spending tend to be cut in situations of debt distress, and at what economy-wide cost? The detailed fiscal incidence work of Nora Lustig (2018) and associates may be valuable here.

• How do local exchange rates, interest rates and cross-border capital flows into low-income commercial borrowers respond to changes in the terms of trade, global interest rates and global growth rates? Understanding these links can improve debt management (e.g., by forecasting the profile of valuation effects on debt ratios) and inform appropriate prudential regulation of banking systems. Are the dynamics very different for countries with flexible exchange rates? Heterogeneous panel time series methods are likely to be useful here, as illustrated for example by Montiel and Pedroni (2019).

• Do LIC DSF risk ratings impact borrowing costs, financial flows and exchange rates? Is this impact stronger for countries with a greater share of short-term and/or commercial debt, or countries with higher debt levels? How does it compare to the impact of private credit-rating scores on these variables among emerging-market countries? Is the risk-rating role of the World Bank and IMF compromised by the lending roles of these institutions, or by the IMF’s role as a manager of debt distress? Should the LIC DSF refrain from issuing definitive ratings for low-income countries, as the IMF does for emerging-market countries?

• What burden-sharing mechanisms can the IMF call upon to involve new creditors in preventative debt restructurings and avoid the destruction of development assets in the resolution of debt distress? How can African countries advance their collective interest in such mechanisms, in a context of global economic pressures and great-power rivalries?

## Footnotes

1 Debt ratios have risen rapidly among the advanced countries as well, with the total public debt of OECD countries nearly doubling in nominal terms from 25 trillion USD in 2008 to a projected 45 trillion in 2018 (OECD, 2018).

2 The average ratio of the present value of external debt to the book value of external debt was 71% for all 44 countries in SSA with data in 2018 and 72% for the 33 SSA HIPCs (World Bank: World Development Indicators).

3 The relevant concepts are IDA eligibility (World Bank, https://ida.worldbank.org/about/borrowing-countries) and PRGT eligibility (IMF). In SSA, the same 39 countries comprise each group in 2020. We exclude Eritrea and Somalia for most purposes due to lack of data.

4 HIPC countries were required to undergo a joint World Bank/IMF debt sustainability analysis at the decision and completion points (see Section 4 below).

5 The HIPC, Enhanced HIPC and MDRI initiatives all adopted uniform treatment of all countries.

6 The valuation term can be very large—see Campos *et al.* (2006).

7 To apply equation [1] to present values, replace $D$ with its present value wherever $D$ appears, and replace ${i}_t$ by the ratio of scheduled repayments to the present value of debt. The grant element in new debt disbursements would then be a component of the primary deficit net of grants, with a negative sign.

8 The predicted path of debt does not have to be explosive to breach these thresholds. A sequence of sufficiently large primary deficits can produce a debt-ratio breach even if $r<g,$ and debt-service breaches can of course occur due to lumpiness in obligated repayments regardless of the time path of the present value of debt.

9 Fund/Bank staff can overrule the risk rating implied by the mechanical signals, particularly when the rating implied by the signals is not clear cut. Lang and Presbitero (2018) note that discretionary adjustments tend to favor countries that are politically aligned with the major shareholders of these institutions.

10 In Easterly’s analysis the distinction between concessional and non-concessional debt is immaterial. The supply of external borrowing at interest rates at or below $r=A$ is assumed to be determined fully by lenders. For the concessional portion of the debt, the present value of future obligations calculated at the interest rate $r$ can be viewed as a market-based loan at rate $r$, with the resulting grant element treated as an outright grant allocated by lenders at the origination of the loan. The grant portion is gone as of the time of debt relief: partly consumed at the time of issue of the debt, and partly stored in the capital stock for future consumption.

11 The average CPIA for this group in 2016–2018 (the latest data) is 3.19.

12 See Arslanalp and Henry (2015) on the favorable efficiency impacts of debt-stock relief under the Brady Plan.

13 Djimeu’s own reading is less favorable than ours, which we find puzzling. He stresses insignificant impacts on growth, for example, but these are present only when total investment is included in the regression. When investment is excluded the impact on HIPC and MDRI relief on growth is large and statistically significant. The appropriate interpretation here seems straightforward: the main effects of debt relief on growth were mediated through investment.

14 In results not shown, the non-concessional share of public and publicly guaranteed external debt in 2012 and the share of domestic debt in total general government debt in 2012 have no predictive power (negligible and highly insignificant coefficients) in regressions for the average risk rating or for either measure of deterioration between 2013 and 2019.

15 A dummy variable for small island states has a positive but statistically significant coefficient when added to the regressions reported in Table 4.

16 Within the IMF, the Buffie *et al.* (2012) approach is known as the ‘Model-Based Framework’ for low-income countries. See IMF Institute (n. d.). For a country application, see Issoufou *et al.* (2014).

17 This was a significant improvement over the average anticipated return of 20% over the previous 40 years, reflecting a significant improvement in the policy environment for growth.
